# Organic-Silica Interactions in Saline: Elucidating the Structural Influence of Calcium in Low-Salinity Enhanced Oil Recovery

**DOI:** 10.1038/s41598-017-10327-9

**Published:** 2017-09-08

**Authors:** J. L. Desmond, K. Juhl, T. Hassenkam, S. L. S. Stipp, T. R. Walsh, P. M. Rodger

**Affiliations:** 1 Department of Chemistry, University of Warwick, Coventry, CV4 7AL UK; 2 Centre for Scientific Computing, University of Warwick, Coventry, CV4 7AL UK; 3 Nano-Science Center, Department of Chemistry, University of Copenhagen, Copenhagen, Denmark; 4 Institute for Frontier Materials, Deakin University, Waurn Ponds, 3216 VIC Australia; 5 Present Address: Department of Chemistry, University of Basel, CH-4056 Basel, Switzerland

## Abstract

Enhanced oil recovery using low-salinity solutions to sweep sandstone reservoirs is a widely-practiced strategy. The mechanisms governing this remain unresolved. Here, we elucidate the role of Ca^2+^ by combining chemical force microscopy (CFM) and molecular dynamics (MD) simulations. We probe the influence of electrolyte composition and concentration on the adsorption of a representative molecule, positively-charged alkylammonium, at the aqueous electrolyte/silica interface, for four electrolytes: NaCl, KCl, MgCl_2_, and CaCl_2_. CFM reveals stronger adhesion on silica in CaCl_2_ compared with the other electrolytes, and shows a concentration-dependent adhesion not observed for the other electrolytes. Using MD simulations, we model the electrolytes at a negatively-charged amorphous silica substrate and predict the adsorption of methylammonium. Our simulations reveal four classes of surface adsorption site, where the prevalence of these sites depends only on CaCl_2_ concentration. The sites relevant to strong adhesion feature the O^−^ silica site and Ca^2+^ in the presence of associated Cl^−^, which gain prevalence at higher CaCl_2_ concentration. Our simulations also predict the adhesion force profile to be distinct for CaCl_2_ compared with the other electrolytes. Together, these analyses explain our experimental data. Our findings indicate in general how silica wettability may be manipulated by electrolyte concentration.

## Introduction

The influence of ionic strength and electrolyte composition on non-covalent adsorption of polar compounds at the aqueous electrolyte/silica interface is important in a wide range of commercial applications^1^, including drug delivery systems^2^, artificial bioglass bone implants^3^ and low-salinity enhanced oil recovery (EOR) from sandstone reservoirs^4, 5^. In low-salinity EOR, there is clear evidence that production from sandstone reservoirs is higher when the water used to sweep the reservoir and maintain pressure has salinity below ~5,000 ppm than when seawater (salinity ~36,500 ppm) is used^6^; this phenomenon is termed the “low-salinity effect” (LSE). Although no additional chemicals are needed to generate the LSE, the response has recently been successfully employed in conjunction with surfactant flooding and polymer flooding^7–9^. Furthermore, EOR techniques in general have increased oil yields in both secondary (*i*.*e*. initial water flooding) and tertiary recovery modes, with the chemical or brine composition of the initial flood influencing the residual oil condition prior to tertiary recovery^5, 9^. In spite of this success, the mechanism of low-salinity EOR is much debated, and over 17 different mechanisms have been proposed to explain it^4, 5^. Several conditions have been identified as conducive to generating the LSE: the presence of polar compounds; the presence of divalent ions; the presence of initial/connate water; the presence of clay; and that the salinity of the injected water should be lower than that of the preceding water^4^. However, satisfying these conditions does not guarantee a low-salinity response. In some cases only a subset of conditions were necessary to observe the LSE, *e*.*g*. the presence of clay may not be required^5, 10–13^. Lager *et al*. proposed that polar organic oil molecules were bonded to the rock surface through bridging by divalent ions and that on injection of low-salinity brine, monovalent ions readily exchanged with the bridging ions in a multicomponent ion exchange (MIE) mechanism, releasing the oil^14^. This migration of divalent ions from the interface is consistent with the prediction of DLVO theory^15^ that electric double layer (EDL) expansion would occur on addition of the low-salinity solution. Indeed, EDL expansion is the basis of many other LSE hypotheses^4^. For example, the greater the thickness of the EDL, the less the negatively charged rock surface is screened, and the greater the repulsion of negatively charged molecules from the surface. MIE and EDL expansion are both closely linked to the most frequently suggested mechanism: wettability modification, *i*.*e*. a change in the affinity of oil or the aqueous phase for the reservoir rock at the molecular level^4, 5^. In summary, the mechanisms by which the LSE works are complex and have, to date, eluded adequate explanation.

Recent studies, using a range of materials to model sandstone, have identified Ca^2+^ ions as important in low-salinity EOR. Mugele *et al*. demonstrated that wettability could be controlled by the concentration of Ca^2+^ ions in solution^16^. The macroscopic contact angle of water on model sandstone surfaces in ambient decane was used to quantify surface wettability. The greater the ionic strength of CaCl_2_ in the aqueous phase, the larger the contact angle and the less water-wet was the mica (*i*.*e*. model sandstone) surface. When small amounts of a model polar hydrocarbon, stearic acid, were added, the contact angles increased significantly from between 1–10° to up to 70°. Near-zero contact angles were observed on silica (another model for sandstone) and for aqueous NaCl and KCl. In spontaneous imbibition experiments using crude oil-brine-Berea sandstone systems, oil-wetness increased with CaCl_2_ concentration, but was not influenced by NaCl concentration^17^. Total organic carbon (TOC) analysis and field emission scanning electron microscopy (FESEM) experiments suggested that the adsorption of benzoic acid to Berea sandstone and silica was enhanced in the presence of Ca^2+^ compared with Na^+^^17^. As described in the MIE mechanism^14^, it was proposed that Ca^2+^ bridges form between negatively-charged organic molecules and the surface. Underwood *et al*. predicted such bridging in a recent molecular dynamics (MD) study of decanoate adsorption at the interface between montmorillonite and mixed NaCl and CaCl_2_ solution^18^. However, the number of bridges did not change with the concentration of background NaCl and it was suggested that the effect did not depend on the concentration of seawater. The chemical force mapping mode (CFM) of atomic force microscopy (AFM) can be used to determine adhesion force. CFM experiments showed that the adhesion of an alkyl-terminated tip to mica was ~23× stronger in aqueous CaCl_2_ than in aqueous NaCl at three different concentrations (10 mM, 100 mM and 1000 mM)^19^. In contrast to previous studies^16, 17^, the adhesion depended on the concentration of NaCl. Adhesion was ~9× greater in 1000 mM solution than in 10 mM solution for *both* CaCl_2_ and NaCl. The concentration of Ca^2+^ has also been observed to affect low-salinity-surfactant EOR^7^. Although oil recovery decreased as expected when the divalent ion/Na^+^ ratio of the injection brine was increased from 0.0175 (with Ca^2+^ as the only divalent) to 0.033 (with Ca^2+^ and a small amount of Mg^2+^), oil recovery in the absence of any divalent ions was *lower* than that with the 0.0175 ratio^7^. The increase in ionic strength as the divalent ion/Na^+^ ratio was increased from 0 to 0.0175 to 0.033 correlated with two opposing effects: a decrease in interfacial tension (IFT), promoting EOR; and an increase in surfactant adsorption, reducing the potential for EOR. The ionic strength effect dominated for the 0.0175 Ca^2+^/Na^+^ ratio. Overall, although water-wetness and/or oil recovery has generally been observed to decrease with increasing CaCl_2_ concentration, there is a lack of atomistic-level insight and the mechanism(s) by which this occurs is/are not understood.

Interfacial ion structure is challenging to probe experimentally, while molecular simulation studies have the potential to provide much needed insight into the structure of the EDL. Lee *et al*. used small angle neutron scattering (SANS) to measure the thickness of thin brine films surrounding silica particles in an *n*-heptane/anionic surfactant mixture^20^. Despite of the large estimated uncertainties, some general trends were identified: as predicted^15^, film thickness decreased with increasing salinity; the larger the monovalent cation, the more sensitive the film thickness to salinity; and films containing divalent cations were more sensitive to salinity. The former two trends are consistent with X-ray photoelectron spectroscopy (XPS) studies^21, 22^ and *ζ*-potential measurements^23, 24^ at the silica/brine interface. The lateral organisation of interfacial ions has been explored using atomic force microscopy (AFM) at the aqueous RbCl/mica interface^25^ and at the aqueous CaCl_2_ and MgCl_2_/gibbsite interfaces^26^. Both studies were conducted in combination with molecular modeling. In the Ricci *et al*. study^25^, short 1 ns molecular dynamics (MD) simulations were used to investigate the stability of different lateral arrangements of adsorbed ions, while in the Siretanu *et al*. study^26^, ion adsorption energies were predicted using density functional theory (DFT). Ricci *et al*. proposed that the lateral ion structuring was driven by the interfacial water structure^25^. The alteration of interfacial water structure with ionic strength is supported by visible infrared sum-frequency generation (vis-IR SFG) spectroscopy experiments at the aqueous NaCl/fused silica interface^27, 28^. Further, a recent MD simulation study of the interface between 0.35 M monovalent chloride and a negatively charged amorphous silica surface predicted that water orientation was strongly dependent on cation identity^29^. This correlation between ionic strength/electrolyte composition and water structure is important because interfacial solvent density can exert a major influence on molecular adsorption^30^. MD simulations have also been used to investigate the impact of ionic strength on EDL structure at the aqueous mixed NaCl - CaCl_2_/montmorillonite interface^31^. Although ion adsorption planes — key to many EDL models (the 0−, *β*− and d-planes *i*.*e*. the average position of inner-sphere complexes, outer-sphere complexes and diffuse swarm species)^32^ — were identified, the location of such planes was independent of ionic strength and ion type. For certain concentrations, MD simulations have predicted the formation of an EDL at *charge-neutral* silica nanoparticles in a range of aqueous electrolytes (NaCl, CaCl_2_ and MgCl_2_)^33^; this observation contrasts with traditional EDL theory, where the presence of a charged surface is a central assumption^34^. In spite of the potential of molecular modeling to elucidate the microscopic structure of the EDL and indeed to give insight into the mechanisms of low-salinity EOR in general, no definitive conclusions have been reached, and to date combined simulation-experiment studies are rare.

Here, we seek to probe EDL expansion and wettability modification, with a particular focus on the influence of the concentration of CaCl_2_ in comparison with that of other aqueous electrolytes present in seawater. A combination of CFM experiments and metadynamics (metaD) simulations was used to study the adsorption of alkylammonium (${R-\text{NH}}_{{\rm{3}}}^{+}$) at the aqueous electrolyte/amorphous silica interface as a function of brine concentration for CaCl_2_, NaCl, KCl and MgCl_2_. Atomistic MD simulations were used to elucidate interfacial ion structure. Amorphous silica is often used as a model for quartz, a mineral that dominates the composition of sandstone and to which crude oil is assumed to be exposed in the pores of the reservoirs^12^. The experiments were conducted at pH 5.5, a pH typical of the formation water in sandstone reservoirs^12^. The surface charge density of the amorphous silica substrate used in simulation, −0.136 C m^−2^, corresponds to the charge state observed at pH 5.5^35^. Although polar compounds are not the most common component of crude oil^36^, their presence has been identified as a key requirement for the observation of low-salinity EOR^4^. Protonated amines (R_3_NH^+^) and carboxylic acids (R-COOH) have also been described as the most reactive organic species towards negatively-charged groups on the reservoir rock surface^37^; but in comparison with R-COOH, the R_3_NH^+^ functionality is underexplored in the field of low-salinity EOR. The ${R-\text{NH}}_{{\rm{3}}}^{+}$ functionality, investigated in this work, is the simplest representation of often more complex nitrogen-containing organic compounds present in crude oil^36^, and of some amine-based surfactants^38, 39^ which might be present in the reservoir from secondary surfactant floods, or indeed, could be present in the water flood for low-salinity-surfactant EOR. To probe the impact of the ion type—specifically Ca^2+^ in comparison with other aqueous electrolytes on the influence of concentration—all aqueous electrolytes were used at the same two concentrations, 0.1 M and 0.3 M, which were accessible by *both* simulation and experiment; 0.1 M is at the lower limit of what could be reliably modeled using atomistic MD simulations. The concentration of Na^+^ in typical seawater (~0.4 M)^40^ is comparable to that of the higher salinity 0.3 M solution.

## Methods

### Experimental Methods

Samples were cleaved from a {001} silicon wafer (Montco Silica inc.) that had developed a thermally oxidized layer of amorphous silica that was 500 nm thick. A MFP-3D Atomic Force Microscope (AFM), developed by Asylum Research, was used to make the adhesion force measurements. Details of force mapping are presented elsewhere^41^. Functionalized tips were used to provide information about the physical behavior of the sample surface. This experimental setup allowed the forces between the organic molecules of the tip and the amorphous silica surface to be probed as a function of solution composition and concentration. All the experiments were made in 0.1 M and 0.3 M solutions of NaCl, KCl, CaCl_2_ or MgCl_2_. All compounds were reagent grade or better and were purchased from Sigma-Aldrich and used as received (NaCl: ACS reagent, ≥99.0%, KCl: AG, ≥99.5%, CaCl_2_: ACS Reagent, ≥99.0%, MgCl_2_: ACS Reagent, ≥99.0%). All solutions were made with freshly deionised water from a Milli-Q column (ultrapure, deionized and resin exchanged to resistivity ≥18.2 M Ωcm) and were adjusted to pH 5.5 with HCl and NaHCO_3_. The mass of acid or base added was negligible with respect to the ionic strength of the solutions.

#### AFM Tip Preparation

The force maps were acquired with Olympus biolever AFM probes that are delivered with cantilevers of two different spring constants: 30 and 6 pN/nm. For this study, the tip functionalization was compatible only with the shorter, stiffer of the two, which had a nominal spring constant of 30 pN/nm. For each experiment, the deflection sensitivity was tested and the individual spring constants were determined more precisely by fitting a Lorentzian function to the thermal spectrum of the cantilever. The spring constant was 30 ± 10 pN/nm in all experiments. To make the ammonium terminated tips, the gold-coated biolevers were treated in a UV-ozone cleaner (Bioforce Nanosciences, Inc., USA) for 20 minutes and then submerged in an ethanol solution of 4.1 mM 11-amino-1-undecanethiol hydrochloride, HS(CH_2_)_11_NH_2_, for at least 24 hours to ensure a complete monolayer coverage.

#### Force Mapping Procedure

The AFM experiments were performed on amorphous silica surfaces that had been UV-ozone cleaned for 20 minutes just before being mounted in the AFM fluid cell. The force maps consisted of 30 × 30 pixels collected over a 5 *μ*m × 5 *μ*m area to provide enough data for determining reasonable mean values. To start the experiments, both tip and sample were immersed in 3 ml of 0.1 M solution and the average adhesion was determined from several sequential force maps. The force maps were taken using the same area on the sample with the same tip so the data could be compared directly. The 0.1 M solution was exchanged with 0.3 M solution and the cycle was repeated for another two sets of data. In the exchange from 0.1 M to 0.3 M solution or from 0.3 M to 0.1 M, about 75% of the solution was removed and replaced with the new solution. For each solution exchange, we sequentially removed and replaced liquid four to six times, to avoid drying of the surface and tip and to preserve the precise location of the tip on the sample. Previous experiments demonstrated that five exchanges produces a solution that is within 1% of the target concentration.

### Computational Methods

#### System Set-Up

The adsorption of methylammonium to the natively-oxidized silicon surface in a range of electrolyte solutions was investigated using metadynamics simulations, carried out using the Gromacs package, Version 4.5.1^42^ with the PLUMED plugin^43^. Methylammonium was modeled as the first step toward a more sophisticated representation of the functionalized AFM tip. The actual molecule that grafted the functional group to the AFM tip was an ${{\rm{NH}}}_{{\rm{3}}}^{+}$-terminated undecane chain, functionalized at the other end with a thiol group, which forms a strong bond to the gold coated AFM tip. Co-operative effects arising from the presence of more than one molecule adsorbing at the interface, such as is likely to happen on a real AFM tip where the radius of curvature is 20–30 nm, were not taken into account. Further simulations of arrays of longer chains, such as undecylammonium, will be the subject of future work.

Eight different electrolyte solutions were modeled: NaCl, KCl, CaCl_2_ and MgCl_2_, each at two different concentrations, 0.3 M and 0.1 M. For each of the 0.3 M and 0.1 M systems, the number of cations was consistent. A sufficient number of cations were introduced to balance the −32*e* surface charge on the amorphous silica substrate (*vide infra*) and then an additional 10 cations and a stoichiometrically equivalent number of anions were added for the 0.1 M system and 50 cations (with anions) for the 0.3 M system. The methylammonium molecule and the electrolyte ions were modeled using the CHARMM27 force-field^44^. Explicit TIPS3P water^45^ was used, along with the force-field describing the natively-oxidized silicon force-field that was reported by Butenuth and co-workers^46^. This force-field was chosen because it had been developed specifically for use with common biomolecule force-fields, such as CHARMM. It should be noted that it is particularly challenging to generate Ca^2+^/water force-fields that capture all of the relevant physical properties. Mamatkulov *et al*. reviewed the performance of a collection of parameter sets and demonstrated that properties, such as the solvation free energy, for the same ion can differ significantly with force-field^47^. Force-field parameters are often optimized from the single ion properties in solution or the crystalline state and, in practice, can fail to reproduce electrolyte thermodynamic properties at finite concentrations. However, we also note that force-fields of this class have been shown to give a highly quantitative representation of Ca^2+^/water systems^48^, to give excellent agreement with experiment for organically-modified calcite/water interfaces^49^, and to shed important new insight into how complex biomolecular compounds can control the behaviour of Ca^2+^/water systems^50, 51^. We conclude that such force-fields are well designed to elicit trends due to cation identity or concentration, but that some caution should be exercised if highly accurate quantitative data is required.

**Figure 1:**
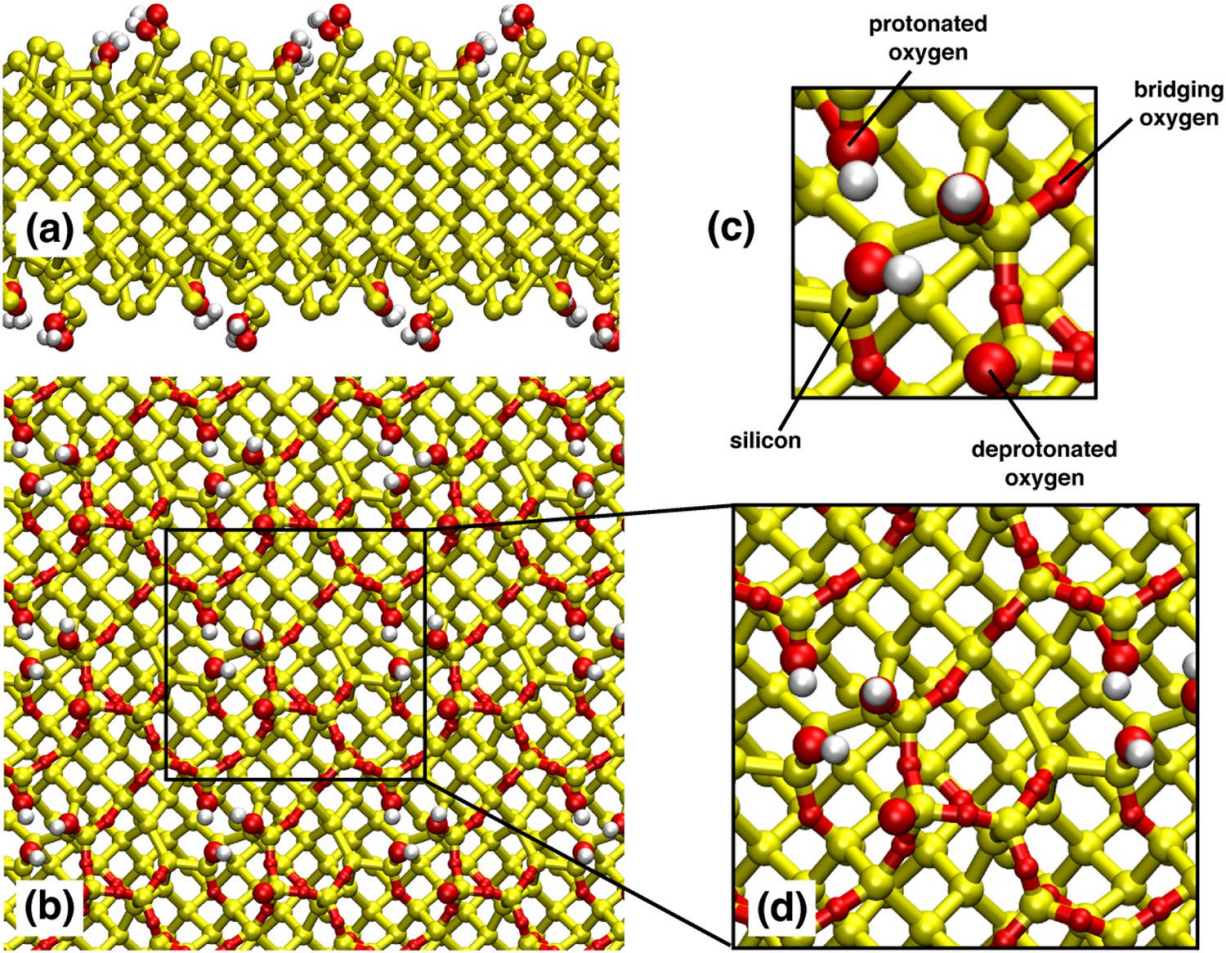
Structure of the model amorphous silica surface used in the MD simulations: (**a**) side on view (**b**) view of surface in the *xy*-plane (**c**) annotated view of a subsection of the surface in the *xy*-plane (**d**) zoomed in view of the surface in the *xy*-plane.

The natively-oxidized silicon substrate was constructed with 2016 atoms, with the surface parallel with the *x* − *y* plane. The top and bottom surfaces of the substrate were identical. The slab consisted of a 4 × 4 super-cell, where each repeat unit featured one deprotonated oxygen (vicinal to a hydroxyl group), three hydroxyl groups, and bridging oxygens. The atomistic structure of the slab is shown in Fig. 1. As for all real mineral surfaces, the atomic-scale structure of amorphous silica is challenging to characterize, because in spite of rigorous surface preparation, exposure to air or solutions leads to adsorption of adventitious carbon^52^. This means that the results from modeling cannot yet be compared directly with data from experiments and, although not the case here, adventitious contamination can lead to problems with the reproducibility of experimental results. All Si and O atoms in the solid slab were immobilized but the H atoms were allowed to move in response to the local forces. The surface charge density of the natively-oxidized silicon substrate was −0.136 C m^−2^, as used by Butenuth *et al*., with −16*e* charge on each of the two surfaces of the slab substrate. This corresponds to the charge state observed at pH 5.5, as used in our experiments^35^. The lateral cell dimensions were 43.488 Å × 43.488 Å. The dimension perpendicular to the cell plane was ~159 Å to provide a volume of electrolyte solution (in the space between the silica slab and its periodic image along the direction perpendicular to the slab surface) was sufficiently large to support bulk electrolyte properties within the center of the inter-slab liquid region.

A time-step of 1 fs was used throughout all the molecular dynamics simulations. The standard method of spherical cutoff was used with the Lennard-Jones interactions (cutoff radius = 10 Å) with long range corrections for energy and pressure^53^. The particle mesh Ewald (PME) method^54^ was used to treat the long-range Coulombic interactions (real-space cutoff = 10 Å). Orthorhombic periodic boundary conditions were applied in all three dimensions. All simulations were performed at 300 K using a Nosé-Hoover thermostat^55, 56^, with a coupling constant of 0.2 ps.

#### Equilibration Simulations

The electrolyte ions were initially positioned randomly in the solution in the interslab space. The water density was then corrected by changing the number of water molecules and repeating the minimization and equilibration calculations until the bulk water density in the centre of the inter-slab space was the same as the water density in an *NPT* simulation of a bulk liquid system (without the surface present) comprising the same composition of ions and water molecules. The number of water molecules in each system differed depending on the electrolyte type and concentration, but was ~9000 in each case (the exact numbers are listed in Supplementary Table S2). MD simulations were run until ion density profiles had equilibrated, between 40–60 ns (see Supplementary Table S1 for the simulation duration in each case). The methylammonium molecule was inserted into the final configuration of each equilibration simulation. No further water density correction was necessary. We then minimized the potential energy of the full system before carrying out further equilibration in the *NVT* ensemble for 200 ps. The final system configuration formed the input file for the metadynamics production runs.

#### Metadynamics Simulations

Well-tempered metadynamics^57, 58^ was used to explore the reaction coordinate (also called collective variable or CV) defined as the perpendicular distance between a static atom of the surface and the nitrogen atom of the methylammonium molecule; given that all of the silica atoms (with the exception of H atoms) were immobilized during the simulations, this is equivalent to driving the perpendicular distance of the N atom from the surface. In the lateral free energy profiles presented herein, the distance was measured such that the baseline of the surface, as defined in the ‘Analysis’ subsection below, was located at *z* = 0; herein we refer to this as the vertical distance. Gaussian hills with a height of 2.9 kJ mol^−1^ and a width of 0.1 Å were deposited every 0.5 ps. The well-tempered ensemble was applied with a bias factor of 10.

In fact, metadynamics was simaltaneously used to explore the in-plane (*x* − *y*) behaviour of the N atom, using the same implementation parameters for *x* and *y* as for the vertical distance (*z*). Given the translational symmetry of the surface, we constrained the simulation to sample just one surface repeat unit of the super-cell by adding constraining walls to the bias potential, thereby considerably reducing the time taken for the calculations to converge. The functional form of the wall was:

1 $${V}_{wall}(s)=\kappa {(s-{{s}}_{{lim}})}^{4}$$

where *κ* is an energy constant, *s* is the value of the CV, and *s*_*lim*_ is a constant denoting the maximum or minimum value that the CV can assume. Lateral *x* − *y* dimensions were constrained to one repeat unit of the slab super-cell, centered on the deprotonated oxygen and at a distance approximately 7 Å from the baseline of the surface (*vide infra*) in the *z* dimension. This also allowed lateral free energy profiles to be calculated as a function of distance from the surface. For this analysis, the simulation cell was divided into slices of thickness 0.05 Å in the *z* dimension and lateral free energy profiles were determined for each of these slices from the 3D metadynamics simulation.

#### Analysis

The surface roughness of the amorphous substrate presented particular challenges to the analysis. The baseline of the surface (*i*.*e*. *z* = 0) was defined by the point of closest approach of the oxygen atoms of water molecules from solution. The simulation cell was divided into slices of thickness 0.1 Å as a function of *z*-distance from the surface, starting with the slice spanning *z* = 0 Å to *z* = 0.1 Å. Surface roughness meant that the initial slices were occupied by smaller volumes of water than those at more distant locations from the surface. To account for this, the surface was divided into a lateral grid, with each of the 144 squares of the grid being of dimension 3.624 Å × 3.624 Å. The number of grid squares occupied by water oxygen atoms was calculated for each of the slices to estimate the solution volume present in each slice. This surface definition did not depend on the concentration or type of ions in the solution. This procedure was carried out using water oxygen position data from the equilibrium MD simulations of the saline solutions.

The silica surface featured deprotonated oxygen sites. Residence times for the cations in the solvation shell of the surface oxygens were calculated by averaging the period of time the ions were closely-associated to these deprotonated oxygen sites throughout the simulation. An ion was defined as closely-associated’ if the distance between its center of mass and that of the deprotonated oxygen site was less than the maximum separation encompassed by the first peak in the deprotonated oxygen-cation radial distribution function (RDF). In the case of CaCl_2_ solutions, chloride ions could bind to calcium ions associated with the deprotonated oxygens, so residence times were calculated by averaging the time the chloride ions were closely-associated with surface-bound cations. A Cl^−^ ion was then defined as closely-associated if the distance between its center of mass and that of the surface-bound Ca^2+^ was less than the maximum separation associated with the first peak in the surface-bound calcium ion-chloride RDF. Relevant RDFs are presented in Supplementary Fig. S1. Standard error, the standard deviation of the mean, was determined and is reported as one *σ*. The standard deviation was calculated using the time periods for which the ions were associated with the deprotonated oxygen and their mean value. This was then used for calculating the standard deviation of the mean.

### Data Availability

The datasets generated during and/or analysed during the current study are available from the corresponding author on reasonable request.

## Results and Discussion

### Chemical Force Mapping Results: Adhesion of the Alkylammonium-Functionalized Tip at the Aqueous Electrolyte/Silica Interface

**Figure 2:**
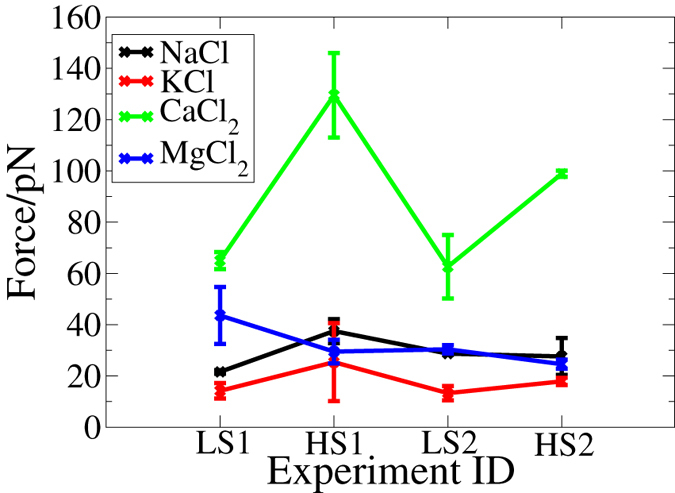
CFM force mapping data: the average adhesion of the ${R-\mathrm{NH}}_{3}^{+}$ functionalized tip during interaction with the amorphous silica substrate during exposure to the solutions at lower salinity (LS), 0.1 M and higher salinity (HS), 0.3 M. 1 refers to the first cycle of experiments and 2 the second cycle.

The adhesion force data determined from CFM experiments are shown in Fig. 2. A clear dependence on concentration was observed for adhesion in the aqueous CaCl_2_. The adhesion force in the higher salinity (0.3 M) CaCl_2_ solution was 50–100% higher than that in lower salinity (0.1 M) CaCl_2_ solution. For MgCl_2_ and the monovalent ions, in contrast, there was little dependence on concentration, although there was a difference in the subtle trends in the adhesion force between MgCl_2_ and the monovalent chlorides. For the monovalent solutions the adhesion force was 10–20 pN larger in 0.3 M solution compared to 0.1 M, whereas, for MgCl_2_, the adhesion force was 5–10 pN larger in 0.1 M solution compared to 0.3 M. Differences in the magnitude of the adhesion force in aqueous CaCl_2_ compared with the other electrolyte solutions were also evident. The adhesion force in the CaCl_2_ solution was 50–100% higher than in the other solutions at 0.1 M salinity and ≥300% higher at 0.3 M salinity.

In previous studies, CaCl_2_ has been identified as having a key influence on the adsorption of organic compounds and the same general trend, *i*.*e*. increasing adhesion with increasing CaCl_2_ concentration, was observed in this work. More specifically, our data can be compared with previous CFM studies of the adsorption of other functionalities at the aqueous electrolyte/model sandstone interface. The adhesion of an alkyl-terminated tip at the aqueous electrolyte/mica interface was dependent on *both* NaCl and CaCl_2_ concentration^19^. A low-salinity response was also observed for a COOH/COO^−^ functionalized tip at an amorphous silica surface in artificial seawater: at high salinity (36,500 ppm), adhesion was 46 pN compared with 33 pN at low salinity (1400 ppm)^12^. These absolute values in adhesion are comparable to those measured in the present work for an alkylammonium functionalized tip in NaCl solution. Na^+^ and Cl^−^ ions dominate the electrolyte composition of seawater and the concentration of Na^+^ at typical seawater salinity is 0.42 M^40^, comparable to the concentrations used in this work (0.1 M and 0.3 M).

### Interfacial Ion Behavior from MD simulation: Differences Between Aqueous CaCl_2_ and the Other Electrolyte Solutions

**Figure 3:**
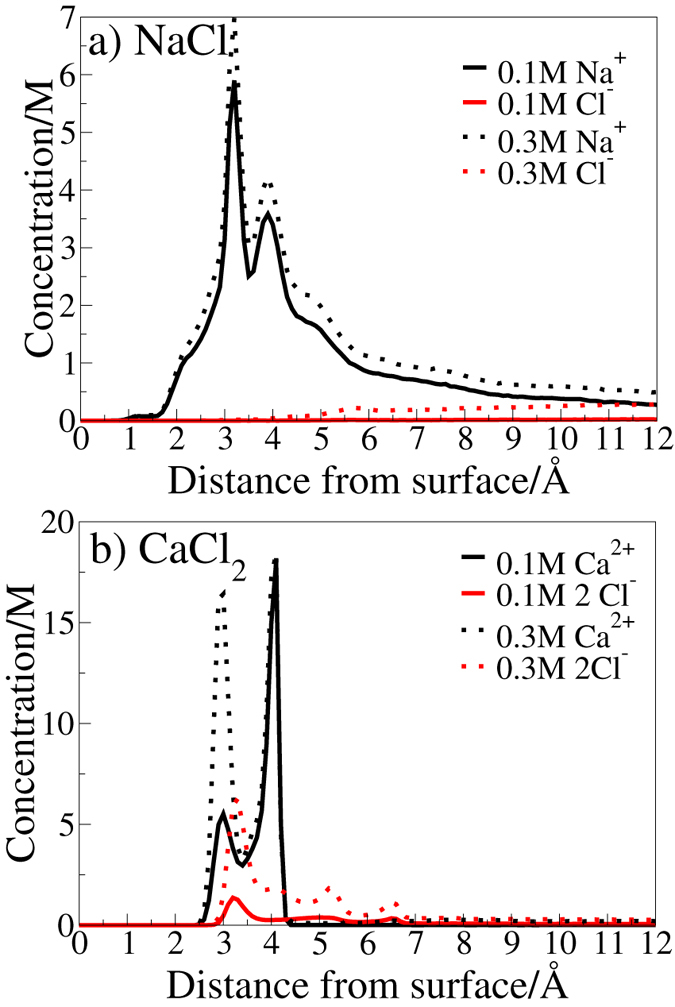
Concentration as a function of distance from the surface for the ions in the NaCl and CaCl_2_ solutions (0.1 M, 0.3 M), determined from the results of MD simulation.

Figure 3 details the ion concentration as a function of vertical distance from the surface for NaCl and CaCl_2_ solutions as calculated from MD simulation. The profiles for KCl and MgCl_2_ (Supplementary Fig. S2) were similar to that of NaCl. The high interfacial cation concentration observed for all of the aqueous electrolytes studied is consistent with other MD simulation results of the aqueous NaCl/silica interface^29, 59^. The electrical double layer (EDL) may be defined as the region that spans from the surface to the point where the cation and anion charge concentrations converge. For practical purposes, we define convergence to have occurred when the magnitude of the cation and anion charge densities remain within 0.01 M of each other. Herein we denote this distance as the Debye length or interfacial region, as determined from our simulations. As can be seen from the interfacial charge density profiles presented in Fig. 3 and Supplementary Fig. S2, and the double layer widths reported in Supplementary Table S3, the EDLs were observed to extend well beyond the layer of cations that is adsorbed onto the silica surface (as indicated by the peaks in the cation interfacial charge densities). This is discussed in more detail hereafter.

#### Double Layer Expansion

For CaCl_2_, NaCl and KCl, the Debye length observed in our simulations was smaller (~25 Å) for the 0.3 M solution than for the 0.1 M solution (~40 Å), while for Mg^2+^ it was ~16.5 Å at both 0.1 M and 0.3 M. DLVO theory predicts that the greater the ionic strength the smaller the Debye length. The ionic strength of divalent chloride solutions is higher than for monovalent chloride solutions. Therefore, the decrease of the Debye length in (1) 0.3 M solution relative to that in 0.1 M solution, and, in (2) divalent MgCl_2_ solution relative to that in the aqueous monovalent chlorides (NaCl and KCl) is consistent with the prediction of DLVO theory^15^. However, the absolute values differ from those calculated using the Debye-Hückel equation (Supplementary Table S3). For MgCl_2_, the Debye length was small and differences between 0.3 M and 0.1 M solution were expected to be negligible. Divalent CaCl_2_ was expected to exhibit similar behavior to MgCl_2_. However, in contrast, the Debye length for CaCl_2_ did not differ from that of the monovalent chlorides at each concentration, and moreover, the spatial distribution of the Cl^−^ counterions was atypical. Unlike the Cl^−^ distributions for the other electrolyte pairs, a high interfacial Cl^−^ concentration was observed within 1 nm of the surface for CaCl_2_. This suggests that DLVO theory is not an effective model for describing electrolyte distributions in CaCl_2_ solution at the amorphous silica interface.

These results are consistent with a previous experimental study, in which EDL expansion was inferred from brine/silica *ζ*-potential measurements (NaCl, KCl and CsCl at 0.001 M and 0.01 M)^23^. In contrast, *β*− and d-planes, key structural features of the EDL, were observed to be independent of ion type *and* ionic strength (0.34–1.83 M) in an MD study of the aqueous mixed NaCl/CaCl_2_/montmorillonite interface^31^. However, at high ionic strengths, such as 0.34–1.83 M, Debye lengths are expected to be small, and hence differences due to inoic strength difficult to determine with any certainty.

In general, the results of the current study verify that lower salinity corresponds to a larger Debye length and so less screening of the negatively charged silica surface: hence to greater repulsion of negatively charged adsorbates from the silica surface, and greater attraction of positively charged adsorbates to the surface. If EDL expansion was the dominant EOR mechanism, then a concentration-dependent difference in the experimentally-determined adhesion of the RNH_3_^+^-functionalized tip would be expected in all of the aqueous electrolytes except MgCl_2_. However, the only concentration-dependent change in adhesion observed experimentally was in aqueous CaCl_2_ and, here, the adhesion was greater in the higher salinity solution (0.3 M compared with 0.1 M). This is the opposite of the trend predicted by the EDL expansion mechanism for a positively-charged adsorbate. Instead, we suggest that this might be explained by another unusual feature of the charge distribution across the interface in aqueous CaCl_2_, namely the presence of a distinct chloride peak between the double Ca^2+^ peak in the interfacial charge density profiles. This feature becomes more prominent at the higher concentration. It is not predicted by DLVO theory, and it is not incorporated into standard double layer models. The increased adhesion force of the ${{\rm{NH}}}_{3}^{+}$ functionalized tip at 0.3 M compared to 0.1 M may be connected to the presence of an increased number of adsorbed Cl^−^ ions, associated with this intervening peak, and thus to an increased electrostatic attraction. This is further elaborated hereafter.

#### Concentration-Dependent Changes in Ion Structure within 1 nm of the Surface

**Figure 4:**
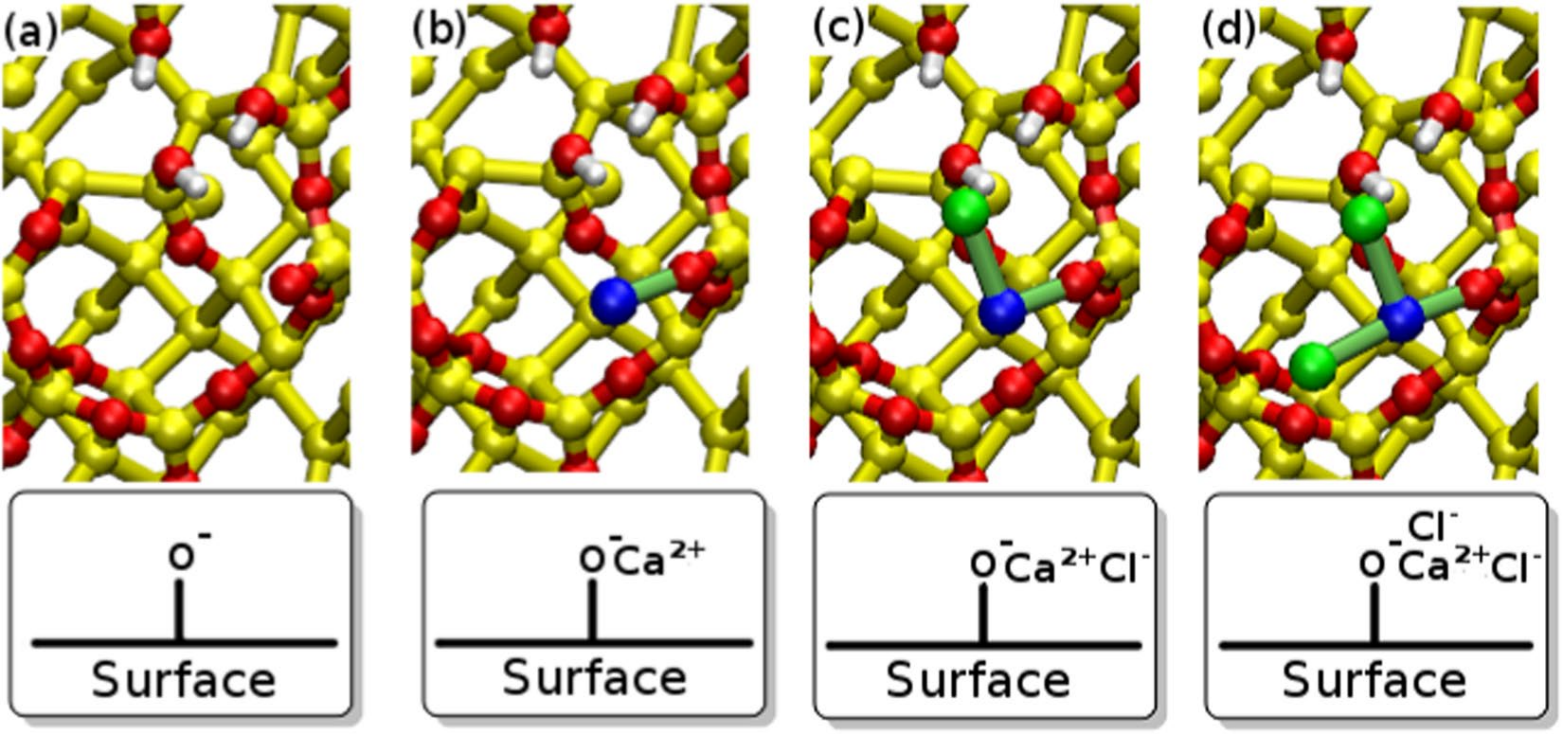
Graphical representations of the different possible states for deprotonated silanols in CaCl_2_ solution. Ca^2+^ ions are represented by dark blue, Cl^−^ ions by green, silicon atoms by yellow, oxygen atoms by red and hydrogen atoms by white. (**a**) Unoccupied O^−^ (Type A) (**b**) Ca^2+^ occupied O^−^ (Type B) (**c**) Ca^2+^Cl^−^ occupied O^−^ (Type C) (**d**) Ca^2+^Cl^−^_2_ occupied O^−^ (Type D).

Further differences in ion structuring between CaCl_2_ solution and the other aqueous electrolytes are apparent in Fig. 3. In addition to the presence of interfacial chloride, the interfacial cation concentration in aqueous CaCl_2_ was found to be more than double that of the other solutions. Moreover, the relative peak heights in the Ca^2+^ vertical density profile changed with concentration. The inner peak (centered at 3 Å) was 25% of the magnitude of the outer peak (centered at 4.1 Å) in 0.1 M solution, whereas in the 0.3 M solution these two peaks were approximately the same height. The underlying physical meaning of the peaks in ion density is explained as follows. In all solutions, interfacial cation structure was dominated by the direct association of cations to deprotonated silanols on the surface, denoted herein as O^−^ (Supplementary Fig. S3). The interfacial chloride ion structure observed in CaCl_2_ was primarily due to the association of Cl^−^ with Ca^2+^ ions that were themselves adsorbed to O^−^ sites on the surface (Supplementary Fig. S3). One consequence of this hierarchy of interactions was the presence of four different classes of deprotonated silanol (O^−^)-based site in CaCl_2_ solution, as illustrated in Fig. 4; herein we refer to these adsorption sites as type A–D, as labeled in Fig. 4.

The presence of such site types is consistent with results from a previous MD simulation study of the aqueous mixed NaCl–CaCl_2_/montmorillonite interface, which reported an affinity of Ca^2+^Cl^−^ ion pairs for the surface^31^. Our structural analysis revealed that the inner peak in Fig. 3b (at 3 Å) was primarily due to Ca^2+^ ions that had associated Cl^−^ ions; these correspond with our site types C and D, depicted in Fig. 4 (see also Supplementary Fig. S4). Ca^2+^ ions involved in the site type C were predominantly located in the regions associated with the inner peak, while those involved in the site type D contributed *only* to the inner peak and not the outer peak. Indeed, the increase in the relative height of the inner peak with concentration correlated with an increase in the proportion of the site types with associated Cl^−^ (types C and D constituted ~25% of all sites for 0.1 M CaCl_2_ compared with a total of ~72% for 0.3 M). A more detailed breakdown of the proportional occupancies of the different site types at 0.1 M and 0.3 M CaCl_2_ is provided in Supplementary Table S4. The outer peak corresponded with Ca^2+^ ions that were adsorbed atop the O^−^ atom whereas the inner peak corresponded with O^−^-adsorbed Ca^2+^ that were buried on the surface (Supplementary Fig. S5). The presence of two characteristic adsorption conformations was further verified by the presence of two peaks in the cos(*θ*) distributions, where *θ* is defined as the angle between the surface normal and r_*Ca*−*O*_, where r_*Ca*−*O*_ = *r*_*Ca*_ − *r*_*O*_ (Supplementary Fig. S4). The corresponding cos(*θ*) distributions for Cl^−^ (Supplementary Fig. S7), where *θ* is defined as the angle made between the surface normal and r_*Cl*−*O*_, where r_*Cl*−*O*_ = *r*_*Cl*_ − *r*_*O*_, featured predominantly one peak at cos(*θ*) ≈ 0 (*i*.*e*. ≈90°). This suggests that the adsorption geometry adopted by Ca^2+^ in the inner peak (*i*.*e*. buried on the surface) facilitated Cl^−^ adsorption at ≈90°).

These observations—that multiple types of ion binding site are found with CaCl_2_ and that their relative preponderance changes with [CaCl_2_]—are extremely interesting in the context of low salinity EOR, and lead us to conjecture a plausible mechanism for the LSE. If oil molecules, such as the positively-charged ${{\rm{RNH}}}_{3}^{+}$ adsorbate, have a greater affinity for the site types that feature associated Cl^−^ (type C and D, Fig. 4) than for the other site types that lack Cl^−^ (namely site types A and B), then the increased proportion of site types C and D at higher [Cl^−^] would generate an increase in the oil-wetness of the interface; conversely, lower [Cl^−^] would reduce the adhesion of organics for the silica, and hence generate enhanced oil recovery. This hypothesis is explored further below, with reference to both the lifetime of the different types of adsorption site, and the free energy for adsorbing methylammonium there too.

#### Electrolyte-Dependent Differences in the Dynamics of Ion-Surface Adsorption

Residence times for the cations in the solvation shell of O^−^ were observed to depend on electrolyte composition (see Supplementary Table S5). Ion-surface residence times were up to three orders of magnitude longer in CaCl_2_ solution (tens of nanoseconds for Ca^2+^ and nanoseconds for Cl^−^) compared with the monovalent cation residence times (tens to hundreds of picoseconds). Given this, the site types A–D, shown in Fig. 4, remained intact for nanoseconds, a relatively long timescale compared to that for the methylammonium adsorption process. While residence times for Mg^2+^ were comparable to Ca^2+^ (at least tens of nanoseconds), the majority O^−^ sites remained vacant throughout the MgCl_2_ simulation, leaving a large preponderance of type A sites with Mg^2+^: 91% for 0.1 M MgCl_2_ and 97% for 0.3 M). It is possible that cations and positively-charged organic molecules, such as ${{\rm{RNH}}}_{3}^{+}$, undergo competitive adsorption at the deprotonated silanol sites. However, if a simple competitive adsorption mechanism had been in operation and the dynamics at this timescale were important, then the adhesion of the ${{\rm{RNH}}}_{3}^{+}$-terminated tip would be expected to be higher in monovalent solutions than in CaCl_2_; instead, actually the reverse trend was observed. Moreover, one implication of the long-lived cation adsorption in CaCl_2_ and MgCl_2_ solutions is that the estimated proportional occupancies of the different adsorption site-types were based on slowly varying data and are likely not fully converged. However, very extensive calculations would be required to determine the equilibrium distributions of these sites with greater accuracy than that presented here; such calculations are planned for a future study.

### Free Energy Landscape for the adsorption of Methylammonium to Silica

**Figure 5:**
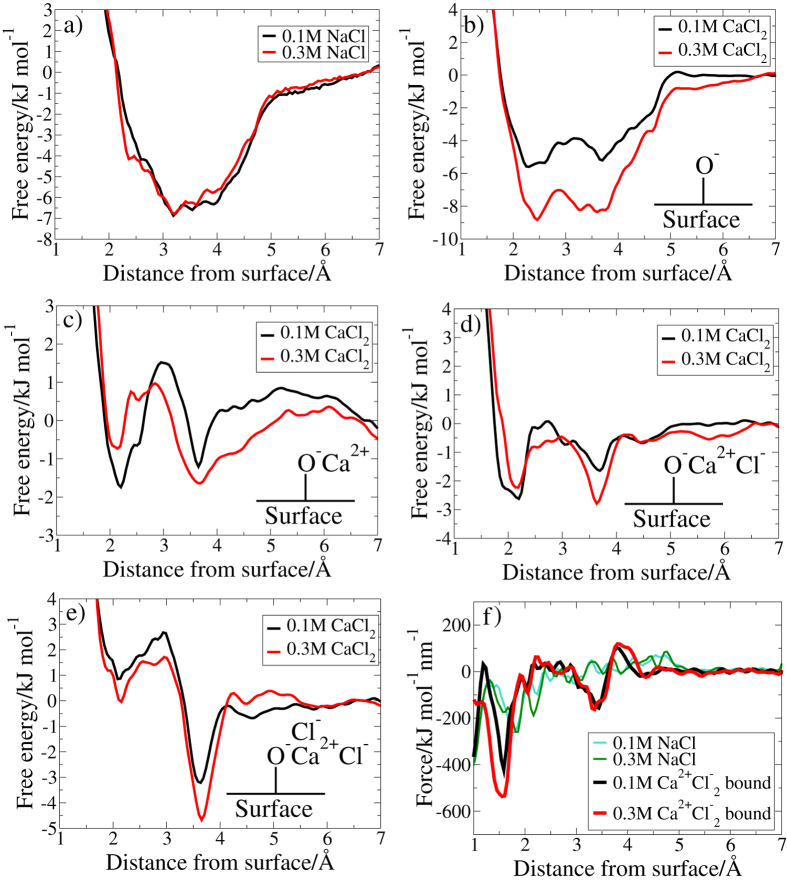
Free energy profiles determined from the MD simulations for the adsorption of N to the silica surface in (**a**) NaCl solution and for different adsorption sites in CaCl_2_ solution: (**b**) unoccupied O^−^ (**c**) Ca^2+^-occupied O^−^ (**d**) Ca^2+^Cl^−^-occupied O^−^ (**e**) ${{\rm{Ca}}}^{2+}{{\rm{Cl}}}_{2}^{-}$-occupied O^−^ (**f**) force curves generated by taking the gradient of N adsorption to silica in solutions of NaCl and CaCl_2_ where ${{\rm{C}}{\rm{a}}}^{2+}{{\rm{C}}{\rm{l}}}_{2}^{-}$ is associated with O^−^.

Metadynamics simulations were used to calculate the free energy of adsorption of the ${{\rm{NH}}}_{3}^{+}$ group of methylammonium (denoted herein as N) as a function of vertical (*z*) distance from the silica surface. The results are presented in Fig. 5 and Supplementary Fig. S5. In all solutions, the key adsorption site for N was the surface O^−^ group. The range of binding scenarios was more complicated in the presence of divalent ions, especially for aqueous CaCl_2_. In NaCl and KCl, cation adsorption and desorption was rapid, leading to a single homogeneous adsorption site which could be characterised by a single adiabatic free-energy curve. However, as discussed above, several different site types (A–D) were identified for divalent cation adsorption, and these were found to persist with lifetime durations that were long compared with the methylammonium adsorption process. To clearly capture the contributions from these different site types, we calculated separate adsorption free energy curves for each of the different site types present for the divalent cation solutions, as shown in(*cf*. Fig. 4). Separation of the adsorption free energy profiles into these different contributions allowed us to test the our wettability hypothesis; namely that wettability—and hence the low salinity response—was linked to changes in the proportional occupancy of site types A–D, and in particular, that adsorption was favored for site types with bound Cl^−^ ions (types C and D). Herein we compare the free energy profiles obtained for each site type, and also present and discuss force-curves obtained from our free energy profiles, in the context of our CFM data.

#### Type A site: influence of electrolyte on methylammonium adsorption at bare O^−^

**Figure 6:**
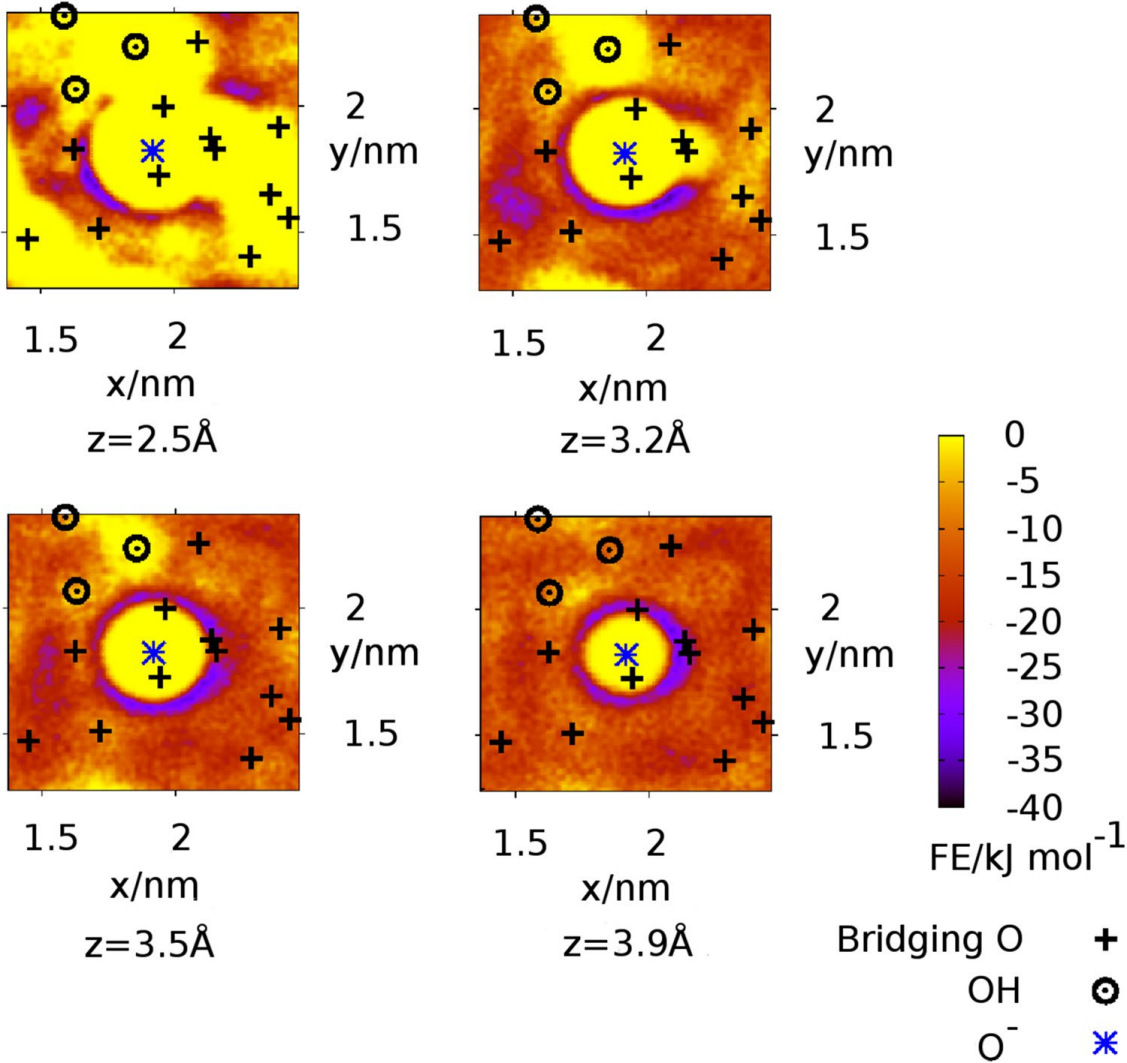
Lateral free energy profiles at a selection of vertical distances from the surface in the 0.1 M NaCl solution. The lateral positions of the surface oxygen sites (*z*-values ≤1.1 Å from the surface) are superimposed on the free energy profiles.

Vertical free energy profiles for the adsorption of N to the surface at type A O^−^ sites are shown in Fig. 5a and b for aqueous NaCl and CaCl_2_. Corresponding data for KCl and MgCl_2_ solutions are similar to those in NaCl, and are presented in Supplementary Fig. S5. In all cases, the free energy well was located between 2–4 Å from the surface. No significant differences (*i*.*e*. no greater than *RT*/2) between 0.1 M and 0.3 M solutions were observed for any of the aqueous electrolytes apart from CaCl_2_. While the general shape of the free energy profiles was similar for CaCl_2_, the free energy well in the 0.3 M CaCl_2_ profile was slightly (~3 kJ mol^−1^) deeper than that for the 0.1 M solution. Moreover, the well-depth of the 0.1 CaCl_2_ profile was slightly shallower than that calculated for the other saline compositions. To better understand the nature of the chemical configurations (*i*.*e*. adsorption of N to the surface) that generated this free energy basin, lateral free energy profiles were calculated at distances every 0.05 Å from the surface (using a *z*-range of 0.05 Å around each distance). Exemplar profiles are presented in Fig. 6 for N in 0.1 M NaCl solution at a selection of distances between 2–4 Å from the surface. At these vertical N-surface separations, the regions of the strongest adsorption were located in a ring around the O^−^ site. These lateral profiles reveal that binding did not occur atop the O^−^ site, and furthermore this lateral distribution was not cylindrically symmetric about the O^−^, indicating that secondary interactions with the rest of the surface were significant. These lateral profiles were characteristic of those seen at comparable vertical N-surface separations in all the other electrolyte solutions. Taken together, these data suggested that N-surface adsorption configurations at site type A did not change with electrolyte solution and, that only in the case of CaCl_2_ did the free energy well-depth vary noticably with solution concentration. The relatively stronger adsorption noted for 0.3 M CaCl_2_ *vs*. 0.1 M for CaCl_2_ was consistent with anticipated stronger attractive force between the positively-charged N site and the increased number of background Cl^−^ ions located within 7 Å of the surface plane. To place these predicted adsorption free energies in context, the adsorption of butyl-ammonium, at various facets of the aqueous fully-hydroxylated *α*-quartz surface, was previously examined using MD simulation^60^. This earlier study found that adsorption free energy of butyl-ammonium ranged from around −2 kJ mol^−1^ to −4 kJ mol^−1^, *i*.*e*. slightly weaker adsorption than is reported here with amorphous silica.

#### Type B site: adsorption of methylammonium at O^−^M^2+^ in divalent cation solutions

**Figure 7:**
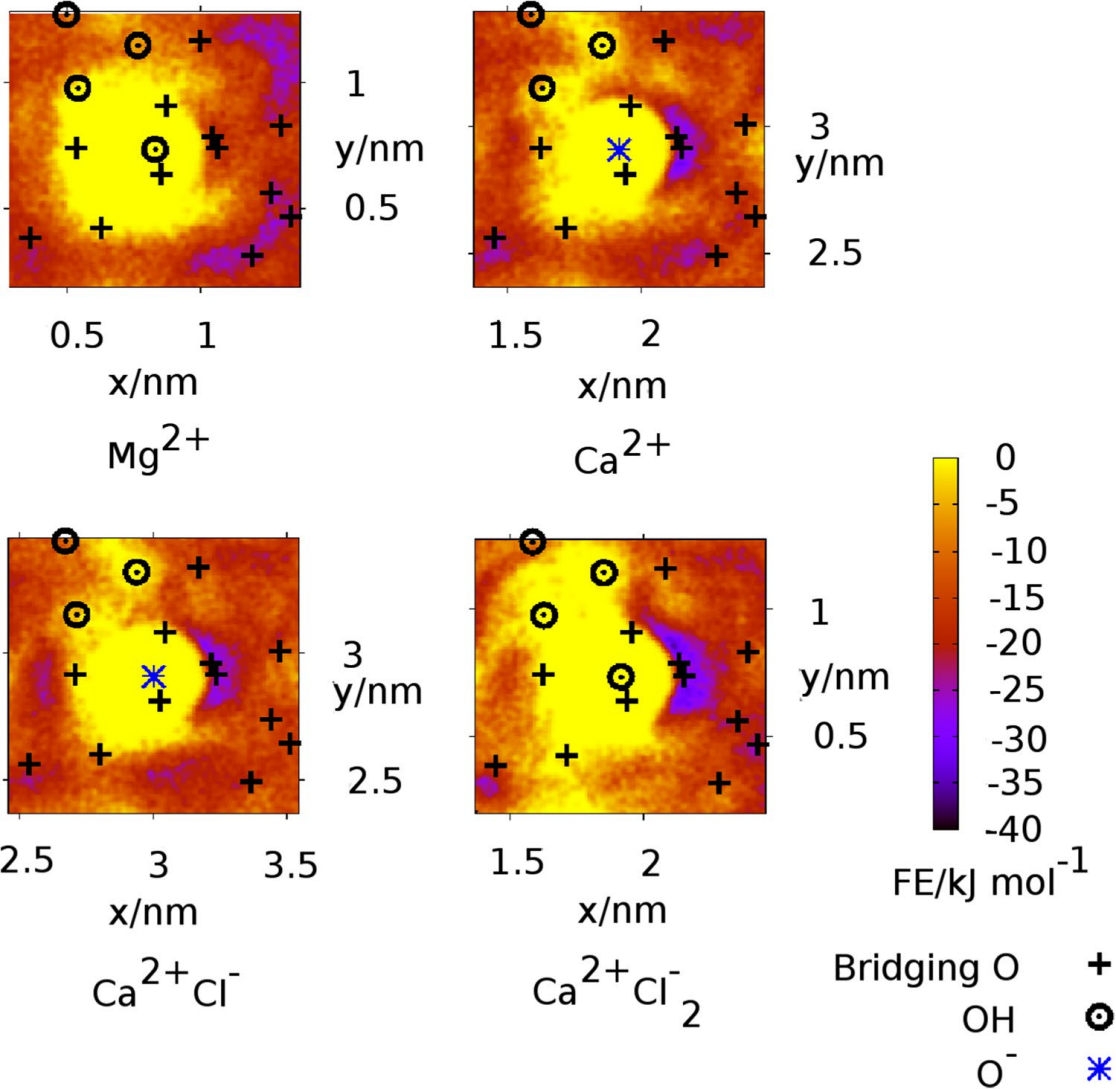
Lateral free energy profiles for N at surfaces with various attachments at O^−^ in 0.1 M aqueous electrolyte at a vertical distance from the surface of 3.7 Å The O^−^Mg^2+^ site was in aqueous MgCl_2_. Any site types that included Ca^2+^ ions were in aqueous CaCl_2_. The data are representative of that in 0.3 M solution. The lateral positions of the surface oxygen sites (*z*-values ≤1.1 Å from the surface) are superimposed on the free energy profiles.

Vertical free energy profiles for the adsorption of N in CaCl_2_ solution to type B surface sites (where the O^−^ site is bound to Ca^2+^, Fig. 4b), are shown in Fig. 5c. The corresponding profile for site type B in MgCl_2_ solution is similar (Supplementary Fig. S5). The predicted adsorption strength for N was slightly weaker (by no more than ~2 kJ mol^−1^) at site type B (where divalent ions were associated with O^−^), compared to site type A (the bare O^−^ site). In fact, binding at type B sites in both divalent cation solutions was predicted to be negligible (~−2 kJ mol^−1^ for CaCl_2_, and −1 kJ mol^−1^ for MgCl_2_). Lateral free energy profiles in the *x*-*y* (horizontal) plane for N, in the aqueous divalent solutions when an O^−^M^2+^ site was present, indicated that the adsorption configurations of N to the surface are dependent on electrolyte. As examples, the lateral free energy distributions for N in 0.1 M CaCl_2_ and 0.1 M MgCl_2_ at 3.7 Å from the surface are shown in Fig. 7. In CaCl_2_ solution the lateral free energy distribution formed a partial ring around the O^−^ site. The area of this lateral adsorption region was less than that observed around the type A site (discussed earlier), where a full ring of low free energy surrounded O^−^ in the lateral profile at this distance from the surface (*e*.*g*. as at 3.9 Å, see Fig. 6). In contrast, the regions of lowest free energy were not located near the O^−^ site in aqueous MgCl_2_, suggesting that the adsorption of N to O^−^ in MgCl_2_ was completely blocked when the site was occupied by Mg^2+^. We conjecture that this is due to the strong water structuring around the Mg^2+^ in solution. The residence time for water in the hydration shell of Mg^2+^ (~2 *μ*s) has previously been reported as several orders of magnitude longer than residence times for water bound to the other ions (ps-ns)^61^. The effective ionic radius of the strongly hydrated Mg^2+^ ion could therefore be greater than that of Ca^2+^, which in turn would result in diminished accessibility of the O^−^ binding site, and the consequent lack of direct N-O^−^ adsorption. The increased interfacial concentration of Mg^2+^ in 0.3 M solution compared to 0.1 M solution (Supplementary Fig. S2), and thus increased proportion of O^−^ sites blocked by Mg^2+^, correlates with the experimentally observed decrease in adhesion force with concentration (Fig. 1).

#### Type C and D sites: adsorption of methylammonium in the presence of associated M^*2+*^ and Cl^−^

Vertical free energy profiles for the adsorption of N to the type C and D sites in CaCl_2_ are shown in Fig. 5c) and d). The type C site corresponds with a O^−^ site coordinated with a single M^2+^ ion, which in turn is associated with a single Cl^−^ (Fig. 4c). In comparison with site type C, the type D site features an additional Cl^−^ associated with the bound M^2+^. As observed for N adsorption in CaCl_2_ at type B sites, these profiles display two well-defined minima: minimum 1 is located 2.1–2.45 Å from the surface, while Minimum 2 is at 3.7 Å. Our data suggest that the free energy well associated with Minimum 2 becomes slightly deeper for site type D compared with site type C (*i*.*e*. as the number of associated Cl^−^ ions at the binding site increases). This increase in binding strength of site type D relative to site type C holds across both concentrations (0.1 M and 0.3 M) of CaCl_2_. Moreover, the well-depth of Minimum 2 varies with CaCl_2_ concentration; binding was a little stronger at higher solution concentrations for both site types C and D.

The corresponding lateral free energy profiles featured a partial ring signifying favorable N adsorption that encircled the O^−^ center in *all* cases. This indicates that Minimum 2 arose mainly from the interaction of N with the O^−^ binding site, regardless of how many Cl^−^ ions were associated. In conjunction with the increase of N adsorption strength indicated in the vertical free energy profiles (Fig. 5), the area of lateral free energy distribution associated with favorable adsorption also increased with the number of associated Cl^−^ ions. In contrast, there was no evidence from our simulations to indicate any adsorption configuration that involved a dominant and direct interaction between the positively-charged N site and the associated Cl^−^. Instead, the lateral free energy distributions suggest that O^−^ accessibility increased as the number of associated Cl^−^ ions increased, indicative of increased repulsion between the two associated Cl^−^ and O^−^ compared with when the adsorption site type involved only one associated Cl^−^ and the absence of such repulsion in the O^−^Ca^2+^ case.

#### Force Curves Derived from Free Energy Profiles Calculated using MetaD

Since average force is the derivative of the free energy with respect to distance, the free energy gradient can be used to estimate the experimental force curves. It should, however, be noted that the free energy curve was generated using simulation data for a single methylammonium molecule, while the experimental AFM tip is functionalised by an array of long-chain alkylammonium molecules. Some quantitative differences are therefore to be expected between simulated and experimental curves. We calculated the free energy gradient (herein referred to as a force curve) from our vertical free energy profiles, for all site types and (where applicable) electrolyte compositions. Exemplar force curves predicted from our simulations are provided in (Fig. 5f). We found that for all site types, the resulting force curves did not change substantially with differing solution concentration. Thus no single site can explain the our experimental CFM findings summarized in Fig. 2. However, the simulated force curves do indicate significant differences between the different adsorption type sites. In particular, the force curve for site type D (found only for the CaCl_2_ solution) was remarkably different compared with all other force curves across both site type and electrolyte composition. This contrast is seen in (Fig. 5f), which shows a strong attractive region spanning 3–4 Å for site type D that was not present in any of the other systems; this was observed for both 0.1 M and 0.3 M solutions. When placed in the context of our earlier analysis, indicating that the fraction of O^−^ sites categorised as type D increased at the higher Cl^−^ concentration, this unique behaviour of the site D force curve provides a viable mechanism to explain the low salinity effect observed in our CFM experiments. Uniquely for CaCl_2_, there are a suite of different CaCl_*n*_ modified O^−^ sites to which the methyl ammonium group can adsorb. These different sites can display different force curves. Whilst the adhesion at each site is insensitive to saline concentration, the relative abundance of the different sites does change with saline concentration. In particular, the site that gives the strongest adhesion (site D) becomes more prevalent at higher CaCl_2_ concentrations, thus generating a higher organic attraction for the silica surface in stronger brines.

## Conclusions

Using an integrated approach that combined chemical force mapping (CFM) experiments with molecular dynamics (MD) simulations, we provided a clear explanation for the molecular-scale origins of the low-salinity effect (LSE), *via* an investigation of the adsorption of a representative organic molecule, positively-charged alkylammonium, at the negatively-charged silica surface, in the presence of electrolyte. To do this, we considered the influence of four different electrolyte compositions (NaCl, KCl, MgCl_2_ and CaCl_2_), at two different concentrations (0.1 and 0.3 M) on the binding of the ammonium moiety. Our CFM experiments showed that adhesion of the alkylammonium to silica was substantially greater in CaCl_2_ compared with the other electrolytes, and moreover, this adhesion was significantly stronger at higher solution concentrations. In contrast, there was no appreciable variation in the alkyammonium adhesion with changes in either solution concentration or electrolyte composition for the other electrolytes. Our MD simulations explained these experimental findings. Specifically, we modeled the four electrolyte solutions in contact with a negatively-charged amorphous silica substrate that contained negatively charged O^−^ surface sites, and calculated the adsorption of positively-charged methylammonium under these conditions. Our simulations revealed four distinct classes of adsorption site on the silica surface, all of which were associated with a de-protonated oxygen surface site. Crucially, we found the relative prevalence of these four site types depended on CaCl_2_ concentration, while remaining broadly invariant to concentration changes in the other electrolytes. The site types most relevant to strong adhesion featured the O^−^ surface site coordinated to a Ca^2+^ in the presence of one or two associated Cl^−^, which become more prevalent at higher CaCl_2_ concentration. Moreover, our simulations predicted that the adhesion force as a function of distance from the surface was remarkably distinct for CaCl_2_ compared with the other electrolyte compositions. Taken together, our force curve predictions and structural analysis from the MD analysis can explain these experimental observations of the LSE. In a broader context, our findings pave the way for understanding and exploiting how surface wettability may be manipulated by electrolyte concentration, which in addition to enhanced oil recovery is important to a wide range of applications, such as biomineralisation, nano-medicine and separation science.

## Electronic supplementary material

Supplementary Information
