# Supplementary material for: Organic-Silica Interactions in Saline: Elucidating the Structural Influence of Calcium in Low-Salinity Enhanced Oil Recovery
Source: Sci Rep. 2017 Sep 8;7:10944. doi: 10.1038/s41598-017-10327-9 (PMC5591284; doi:10.1038/s41598-017-10327-9)
Supplement: Supplementary file 1 — Supplementary Information [file 41598_2017_10327_MOESM1_ESM.pdf]

Supplementary Information for: Organic-Silica Interactions in Saline: Elucidating the Structural Influence of Calcium in Low-Salinity Enhanced Oil Recovery

J. L. Desmond[1,2,5*], K. Juhl[3], T. Hassenkam[3], S. L. S. Stipp[3], T. R. Walsh[4*], and P. M. Rodger[1,2†]

[1]Department of Chemistry, University of Warwick, Coventry CV4 7AL, UK
[2]Centre for Scientific Computing, University of Warwick, Coventry CV4 7AL, UK
[3]Nano-Science Center, Department of Chemistry, University of Copenhagen, Denmark
[4]Institute for Frontier Materials, Deakin University, Waurn Ponds, 3216 VIC, Australia
[5]Current Address: Department of Chemistry, University of Basel, CH-4056 Basel, Switzerland
[†]Died March 23, 2017.
[*]tiffany.walsh@deakin.edu.au
[*]Jasmine.Desmond@unibas.ch

Contents

Supplementary Table S1: Summary of MD simulation times for each electrolyte system.

Supplementary Table S2: Summary of the number of water molecules used in each simulation system.

Supplementary Table S3: Summary of the thicknesses of electric double layers (EDLs) for the different aqueous electrolytes.

Supplementary Table S4: Average percentage occupancy of Site Types A–D in $CaCl_2$ solution.

Supplementary Table S5: Summary of cation/anion residence times at deprotonated O^- surface sites, for all four saline compositions.

Supplementary Figure S1: Cation-deprotonated oxygen RDFs and surface-bound cation-anion RDFs.

Supplementary Figure S2: Ion concentration profiles as a function of distance from the silica surface.

Supplementary Figure S3: Contribution to the surface density profiles from ions represented by significant peaks in O^--M rdfs and O^-Ca^{2+}-Cl^- rdfs.

Supplementary Figure S4: Distributions of cos(θ) where θ is the angle made between the surface normal and r_{Ca-O}, where $r_{Ca-O} = r_{Ca} - r_O$.

Supplementary Figure S5: Vertical free energy profiles for the adsorption of N to silica in aqueous KCl and $MgCl_2$ (unoccupied O^- and Mg^{2+}-occupied O^-)

Supplementary Figure S6: Lateral distributions of Ca^{2+} ions within horizontal layers centered at the peaks in cation z-density.

Supplementary Figure S7: Distributions of cos(θ) where θ is the angle made between the surface normal and r_{Cl-O}, where $r_{Cl-O} = r_{Cl} - r_O$

Electrolyte Solution	Simulation Time/ns	Equilibration Time/ns
0.1 M NaCl	170	50
0.3 M NaCl	180	60
0.1 M KCl	150	50
0.3 M KCl	120	40
0.1 M $CaCl_2$	180	60
0.3 M $CaCl_2$	180	40
0.1 M $MgCl_2$	180	60
0.3 M $MgCl_2$	120	40

Supplementary Table S1. Summary of the total simulation time and equilbration time for each mineral/electrolyte system.

Electrolyte Solution	Number of Waters
0.1 M NaCl	9028
0.3 M NaCl	9009
0.1 M KCl	8997
0.3 M KCl	8959
0.1 M $CaCl_2$	9039
0.3 M $CaCl_2$	9017
0.1 M $MgCl_2$	9044
0.3 M $MgCl_2$	9035

Supplementary Table S2. number of water molecules used in each simulation.

Electrolyte Solution	Width (MD)/Å	Width (Debye-Huckel equation)/Å
0.1 M NaCl	42.7	9.6
0.3 M NaCl	24.9	5.6
0.1 M KCl	39.8	9.6
0.3 M KCl	24.2	5.6
0.1 M $CaCl_2$	41.5	5.6
0.3 M $CaCl_2$	25.4	3.2
0.1 M $MgCl_2$	16.6	5.6
0.3 M $MgCl_2$	16.4	3.2

Supplementary Table S3. Thickness of the electical double layer (EDL) calculated from molecular dynamics simulations for the different electrolyte solutions. The thickness of the EDL is defined as the distance between the silica surface and the point beyond which the cation and anion charge densities converge. For comparison, the EDL thickness has also been calculated according to the Debye-Hückel equation (ref: Kohonen *et. al.*, *Langmuir*, 2000, *16*, 5749).

Site type	Concentration/M	Proportion/%
O^-	0.1	28.6
Type A	0.3	4.7
O^-Ca^{2+}	0.1	46.0
Type B	0.3	28.1
$O^-Ca^{2+}Cl^-$	0.1	24.1
Type C	0.3	51.9
$O^-Ca^{2+}Cl_2^-$	0.1	1.3
Type D	0.3	19.9

Supplementary Table S4. Time averaged proportion (given as a percentage) of occupancy of different site types A–D in $CaCl_2$ solution.

Solution	Concentration/M	Ion	Cutoff/nm	r_t/ns
NaCl	0.1	Na^+	0.3	0.346 ± 0.002
	0.3	Na^+	0.3	0.339 ± 0.002
KCl	0.1	K^+	0.35	0.038 ± 0.0001
	0.3	K^+	0.35	0.037 ± 0.0001
$CaCl_2$	0.1	Ca^{2+}	0.27	57.2 ± 2.1
	0.3	Ca^{2+*}	0.27	> 40.0
	0.1	Cl^-	0.284	5.1 ± 3.5
	0.3	Cl^-	0.284	2.9 ± 3.4
$MgCl_2$	0.1	Mg^{2+*}	0.25	> 60.0
	0.3	Mg^{2+*}	0.25	> 40.0
	0.1	Mg^{2+}	0.5	0.044 ± 0.01
	0.3	Mg^{2+}	0.5	0.037 ± 0.005

Supplementary Table S5. Residence time (r_t) for cations adsorbed to the deprotonated silanols and for anions associated with adsorbed cations. An ion was defined as adsorbed if the distance between its center of mass and that of the deprotonated oxygen site was less than the cutoff *i.e.* the separation corresponding to the first peak in the deprotonated oxygen-cation radial distribution function (rdf). An additional larger cutoff was used for hydrated, associated Mg^{2+}. For $CaCl_2$, Cl^- ions were defined as adsorbed if the distance between its center of mass and that of the surface-adsorbed Ca^{2+} was less than the cutoff *i.e.* the separation corresponding to the first peak in the surface adsorbed Ca^{2+}-Cl^- radial distribution function (rdf).

*Ions that remained bound for the entire simulation. Standard errors are shown in parantheses.
Cl^- ions in simulations for NaCl, KCl and $MgCl_2$ solutions rarely associated so residence times were not determined.

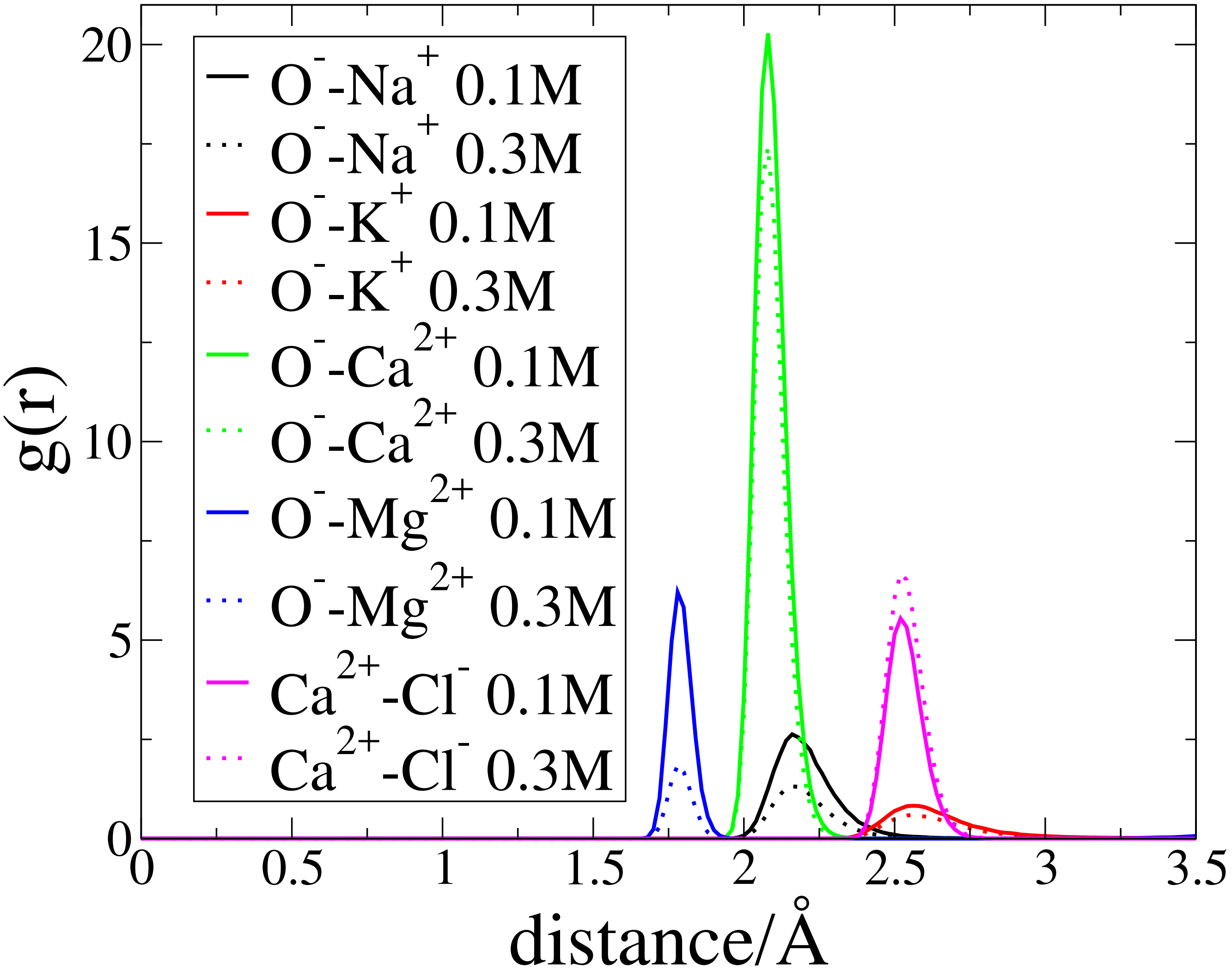

Supplementary Figure S1. RDFs between the cation and silica deprotonated oxygen, and RDFs between the surface-bound cation-anion pair.

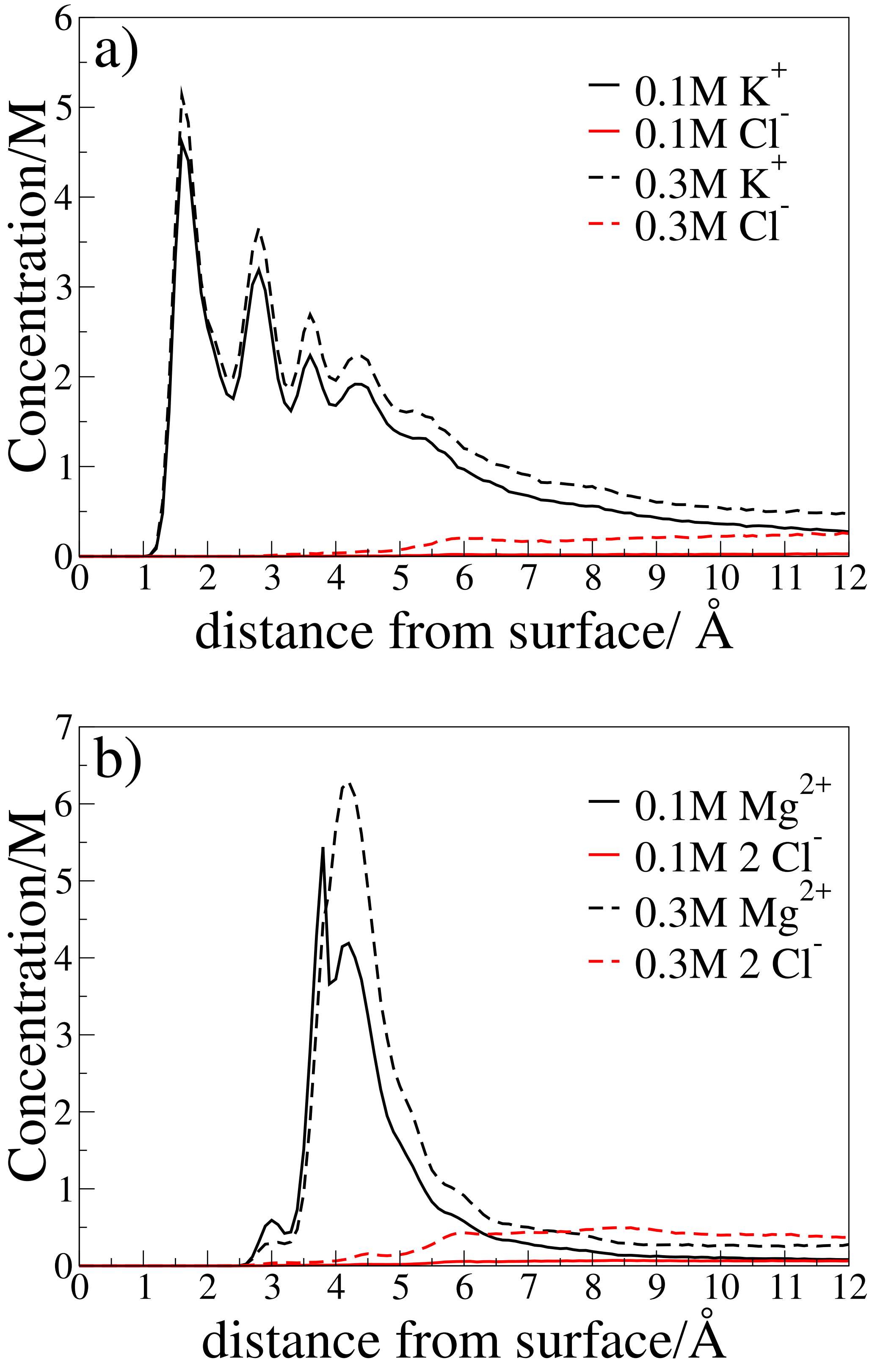

Supplementary Figure S2. Concentration as a function of distance from the surface for the ions in the KCl and $MgCl_2$ solutions (0.1 M and 0.3 M).

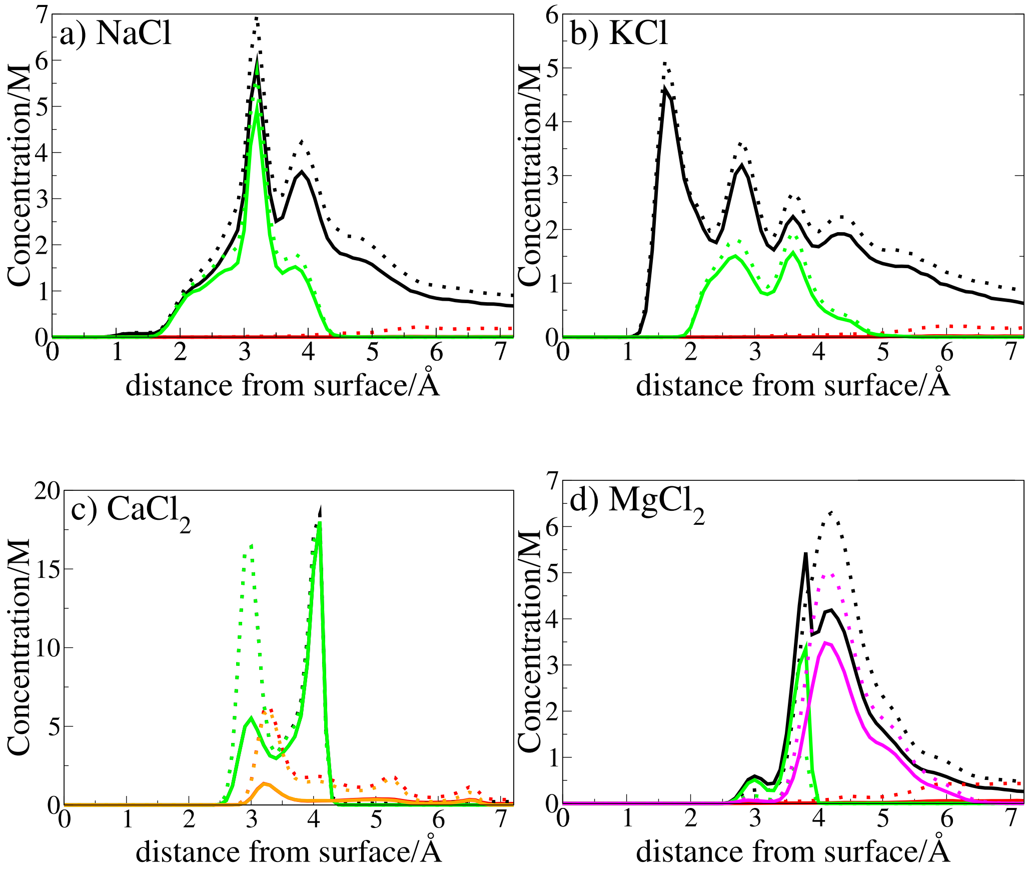

Supplementary Figure S3. Contribution to the surface density profile arising from cations associated with the first peak of the deprotonated silanol-cation rdfs (Supplementary Fig. S1), CSDP(O^--Mrdfp1). Analogous data is given for the second rdf peak for $MgCl_2$ (CSDP(O^--Mg^{2+}rdfp2)) and the first rdf peak of the O^--associated calcium-chloride rdf (CSDP(O^-Ca^{2+}-Cl^-rdfp1)). The complete surface density profiles are reproduced from Fig. 3 and Supplementary Fig. S2 to aid comparison. As in Fig. 3 and Supplementary Fig. S2, cation concentration is represented by black and anion concentration by red. Green represents the CSDP(O^--Mrdfp1) and, for $MgCl_2$, pink is the CSDP(O^--Mg^{2+}rdfp2). Orange represents the CSDP(O^-Ca^{2+}-Cl^-rdfp1). The solid lines represent 0.1 M solution and the dashed lines 0.3 M. In part c) of this diagram, the CSDP(O^--Ca^{2+}rdfp1) overlays the complete Ca^{2+} density profile and the CSDP(O^-Ca^{2+}-Cl^-rdfp1) the Cl^- density profile for both 0.1 M and 0.3 M.

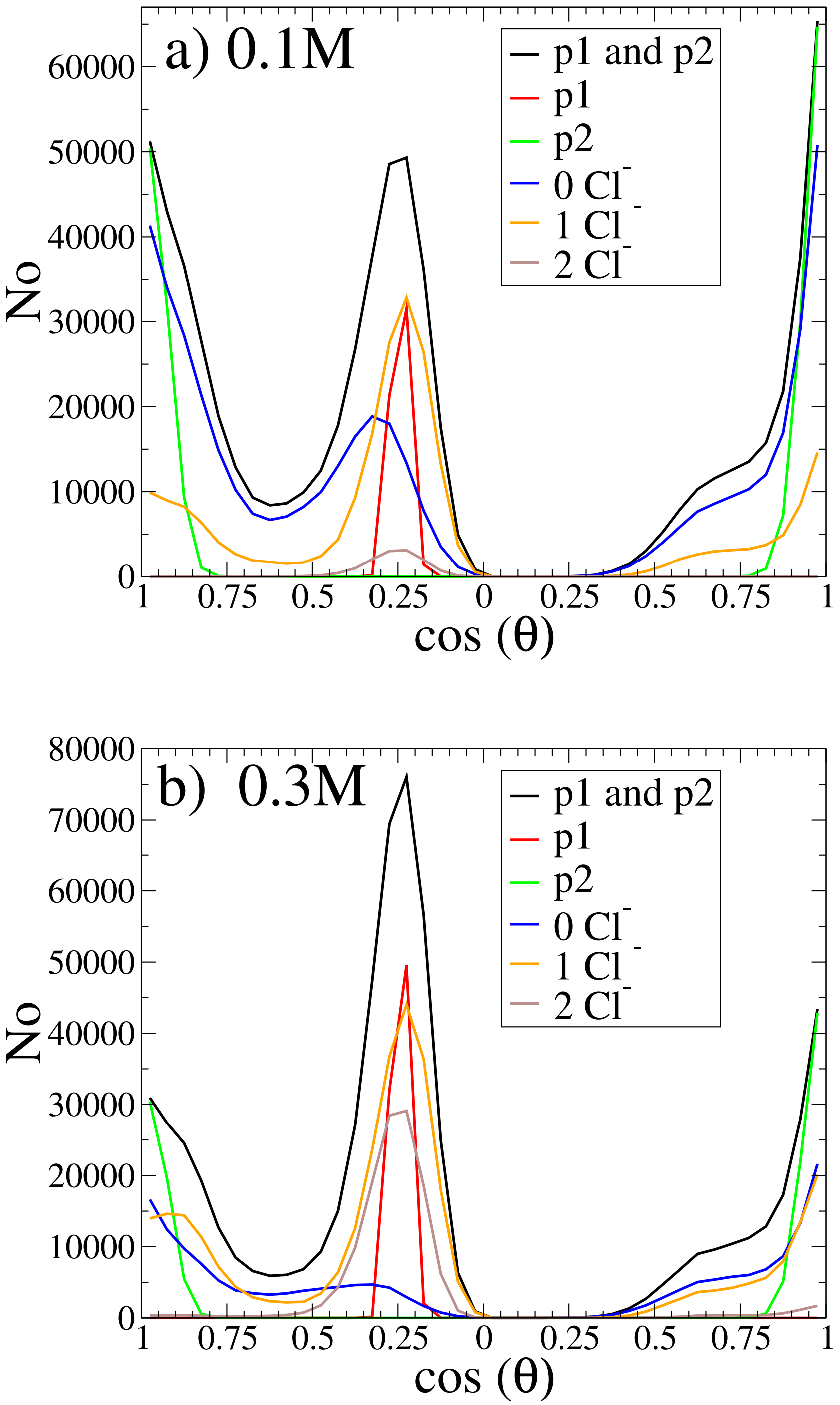

Supplementary Figure S4. Distributions of cos(θ) where θ is the angle made between the surface normal and r_{Ca-O}, where $r_{Ca-O} = r_{Ca} - r_O$ for 0.1 M and 0.3 M solutions. 'p1 and p2' represents cos(θ) for surface-adsorbed Ca^{2+} ions within the entire span of z-values from the beginning of peak 1 (p1) - which is centered at 3.0 Å from the surface - to the end of peak 2 (p2) - which is centered at 4.1 Å from the surface - in Fig. 3 (*i.e.* 2.2-4.4 Å from the surface). 'p1' represents cos(θ) for surface-adsorbed Ca^{2+} ions in the central z-range of peak 1 in Fig. 3 (*i.e.* 2.9-3.1 Å from the surface). 'p2' represents cos(θ) for surface-adsorbed Ca^{2+} ions in the central z-range of peak 2 in Fig. 3 (*i.e.* 4.0-4.2 Å from the surface). '0 Cl^-', '1 Cl^-' and '2 Cl^-' represent cos(θ) for surface-adsorbed Ca^{2+} ions with 0, 1 and 2 associated Cl^- ions, respectively.

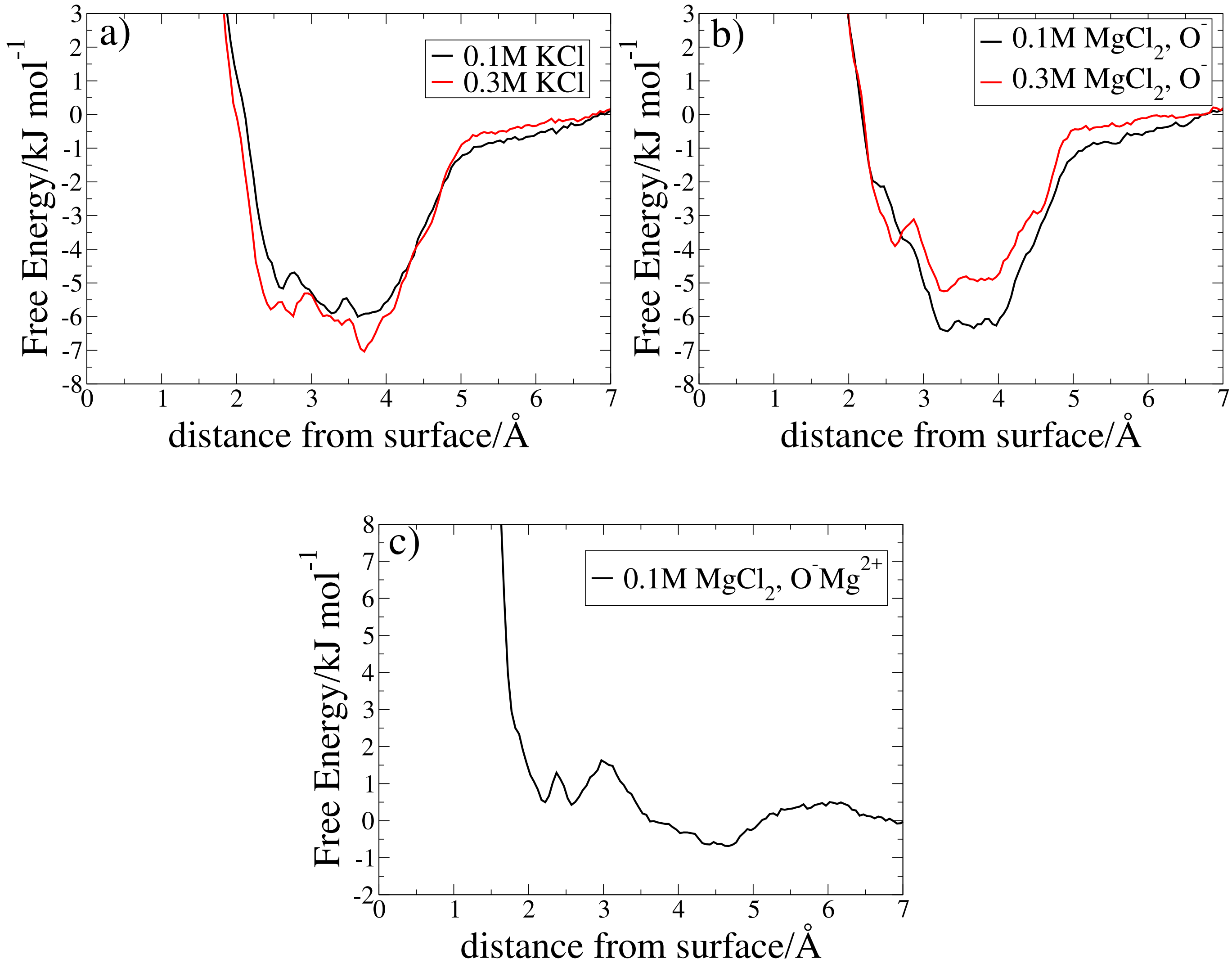

Supplementary Figure S5. Free energy profiles for the adsorption of N to silica in different aqueous electrolytes (a) KCl (b) $MgCl_2$ solution, unoccupied O^- (c) 0.1 M $MgCl_2$ solution, Mg^{2+} occupied O^-

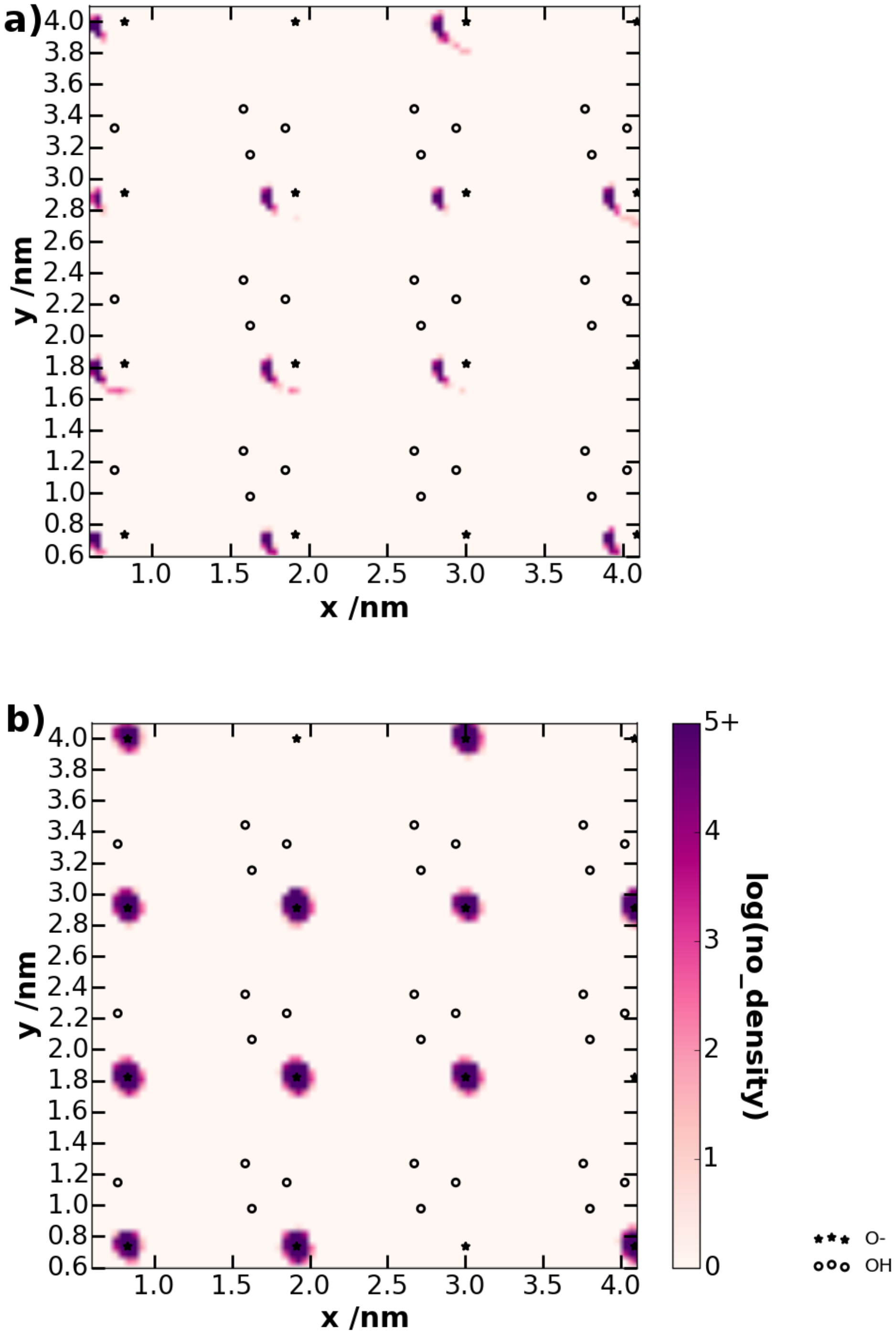

Supplementary Figure S6. ρ_{Zmax}(xy) (lateral distribution of cations found within a horizontal layer of thickness 0.1 Å and centred on the peaks in density as shown in Figure 3b for $CaCl_2$ solution (0.1 M): a) at 3.0 Å from the surface (Ca^{2+} peak 1) b) at 4.1 Å from the surface (Ca^{2+} peak 2). The units of the number density are arbitrary and the same in all lateral profiles throughout this section.

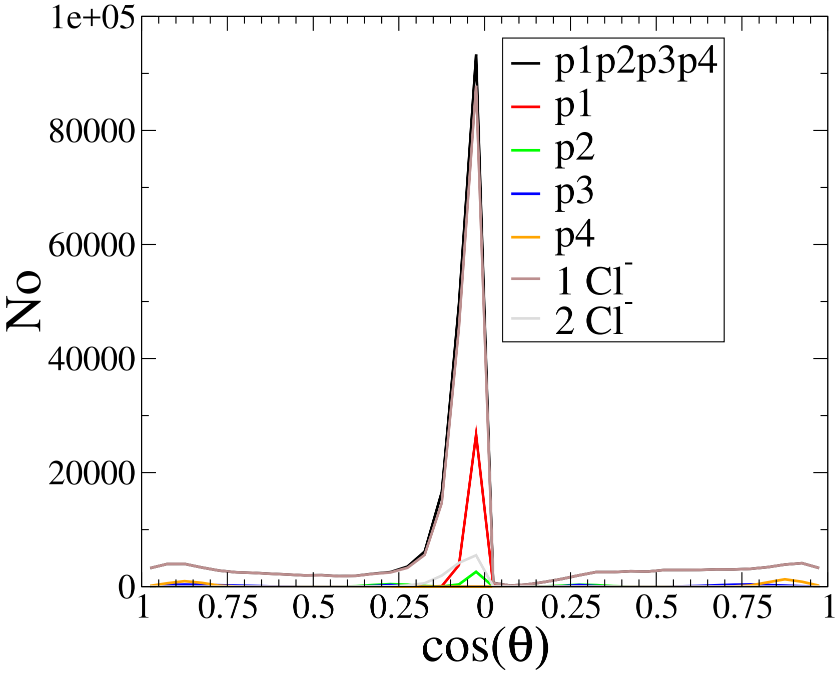

Supplementary Figure S7. Distributions of cos(θ) where θ is the angle made between the surface normal and r$_{Cl-O}$, where r$_{Cl-O} = r_{Cl} - r_O$ for 0.1 M solution. 'p1p2p3p4' represents cos(θ) for surface-adsorbed Ca^{2+} ions within the entire span of z-values from the beginning of peak 1 (p1) - which is centered at 3.3 Å from the surface - to the end of peak 4 (p4) - which is centered at 6.5 Å from the surface - in Fig. 3 (*i.e.* 1.6-6.9 Å from the surface). 'p1' represents cos(θ) for surface-adsorbed Cl^- ions in the central z-range of peak 1 in Fig. 3 (*i.e.* 3.2-3.4 Å from the surface). 'p2' represents cos(θ) for surface-adsorbed Cl^- ions in the central z-range of peak 2 in Fig. 3 (*i.e.* 4.0-4.2 Å from the surface). 'p3' represents cos(θ) for surface-adsorbed Cl^- ions in the central z-range of peak 3 in Fig. 3 (*i.e.* 5.1-5.3 Å from the surface). 'p4' represents cos(θ) for surface-adsorbed Cl^- ions in the central z-range of peak 4 in Fig. 3 (*i.e.* 6.4-6.6 Å from the surface). '1 Cl^-' and '2 Cl^-' represent cos(θ) for surface-adsorbed Cl^- ions where 0 and 1 further Cl^- ions are also associated to the same surface-adsorbed Ca^{2+} ion, respectively.
